# Chemical acylation of an acquired serine suppresses oncogenic signaling of K-Ras(G12S)

**DOI:** 10.1038/s41589-022-01065-9

**Published:** 2022-07-21

**Authors:** Ziyang Zhang, Keelan Z. Guiley, Kevan M. Shokat

**Affiliations:** 1 Department of Cellular and Molecular Pharmacology and Howard Hughes Medical Institute, University of California, San Francisco, CA USA; 2 Present Address: Department of Chemistry, University of California, Berkeley, CA USA

**Keywords:** Cancer, Small molecules, Cell signalling, X-ray crystallography

## Abstract

Drugs that directly impede the function of driver oncogenes offer exceptional efficacy and a therapeutic window. The recently approved mutant selective small-molecule cysteine-reactive covalent inhibitor of the G12C mutant of K-Ras, sotorasib, provides a case in point. *KRAS* is the most frequently mutated proto-oncogene in human cancer, yet despite success targeting the G12C allele, targeted therapy for other hotspot mutants of *KRAS* has not been described. Here we report the discovery of small molecules that covalently target a G12S somatic mutation in K-Ras and suppress its oncogenic signaling. We show that these molecules are active in cells expressing K-Ras(G12S) but spare the wild-type protein. Our results provide a path to targeting a second somatic mutation in the oncogene *KRAS* by overcoming the weak nucleophilicity of an acquired serine residue. The chemistry we describe may serve as a basis for the selective targeting of other unactivated serines.

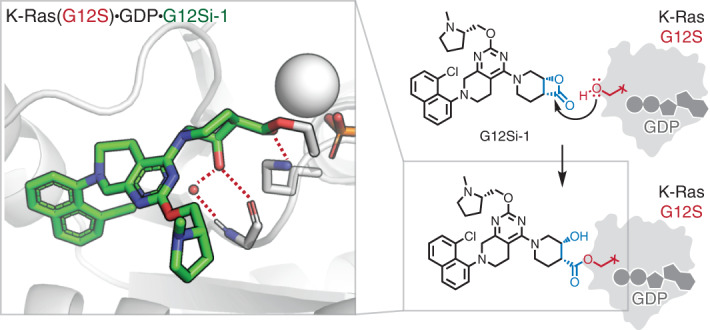

## Abstract (web summary)

The discovery of a strained β-lactone electrophile that covalently targets a KRAS G12 somatic mutation and acylates the mutant serine to suppress oncogenic signaling.

## Main

Mutations in the *KRAS* proto-oncogene are the most frequently observed genetic lesion in human cancer and are estimated to account for one million deaths every year worldwide^1,2^. The *KRAS* gene encodes a small GTPase that controls pro-growth signaling in cells by cycling between the GTP-bound and GDP-bound states. Hotspot mutations on *KRAS* compromise GTP hydrolysis^3–5^ or facilitate nucleotide exchange^5,6^, leading to prolonged and enhanced signaling transduction. Direct, mutant selective inhibition of oncogenic K-Ras mutants presents ideal therapeutic opportunities that have been pursued with various modalities including small molecules^7–19^, cyclic peptides^20–22^, therapeutic macromolecules^23–28^ and targeted protein degraders^29,30^, among others^31^. Discovery of the allosteric Switch-II pocket (S-IIP) and identification of covalent ligands of K-Ras(G12C) demonstrated that K-Ras is a druggable target, whose inhibition confers marked clinical benefit. To date, one such inhibitor (sotorasib) has received approval by the U.S. Food and Drug Administration, and at least five additional drug candidates are under clinical investigation^32^. Despite this success, covalent drugs that target other noncysteine hotspot mutants of K-Ras remain to be developed owing to the low nucleophilicity of residues other than cysteine. The Gly-12 codon in *KRAS* (GGT) is a site of multiple base changes observed in cancer. The smoking-induced transversion mutation (c.34G>T) to produce the TGT codon gives rise to the druggable G12C oncogene. The nonsmoking-related transition mutation (c.35G>A) at the second position produces the GAT codon which is the most common G12D allele. The c.34G>A transition at the first position produces the serine codon (AGT). Here we present small molecules that irreversibly bind to K-Ras(G12S), a hotspot mutant accounting for 4.4% of all *KRAS* mutations^33^. To covalently target a serine residue, we were guided by a family of natural products that possess a strained β-lactone (for example, salinosporamide A, omuralide) and inhibit the 20S proteasome by forming a covalent bond with the catalytic threonine (Thr1)^34,35^. We show that K-Ras ligands that possess a β-lactone are potent electrophiles that bind to K-Ras(G12S) in the S-IIP and rapidly acylate the mutant serine residue, in much the same way that acrylamides are used to target the much more nucleophilic cysteine side chain. We have identified molecules that fully engage K-Ras(G12S) in cells and suppress its oncogenic signaling without affecting its wild-type counterpart.

## Results

### K-Ras(G12S) is an oncogenic driver with GTPase activity

**Fig. 1:**
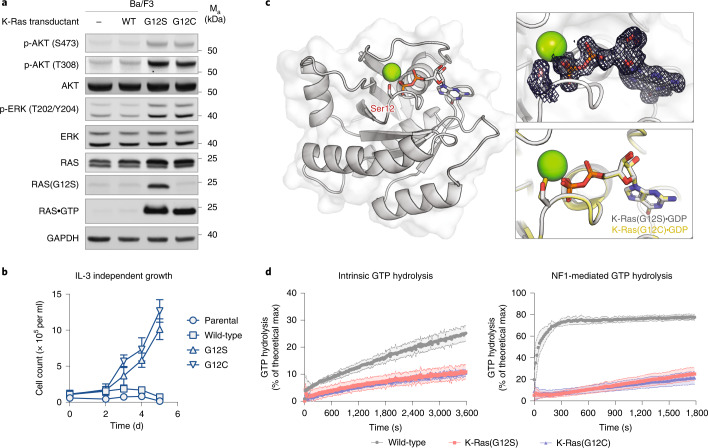
K-Ras(G12S) is an oncogenic driver with intrinsic GTPase activity. **a**, Immunoblot of Ba/F3 cells expressing wild-type K-Ras (WT), K-Ras(G12S) or K-Ras(G12C). IL-3 was removed from the culture media 10 min before cells were lysed and analyzed. Data are representative of two experiments using independently generated Ba/F3 transductants. For gel source data, see Supplementary Information. **b**, Growth of Ba/F3 transductants in the absence of IL-3 (*n* = 3). Data are representative of two experiments using independently generated Ba/F3 transductants. Error bars represent s.d. of three technical replicates. **c**, X-Ray crystal structure of K-Ras(G12S)•GDP. Insets depict the 2*F*_o_ – *F*_c_ map for the mutant serine and GDP (1.0*σ*, gray mesh) and the superimposed structures K-Ras(G12S)•GDP (this structure, gray) and K-Ras(G12C)•GDP (PDB: 4L8G, yellow). For X-ray crystallography data collection and refinement statistics, see Supplementary Table 1. **d**, Intrinsic and NF1-mediated single-turnover GTPase activity of wild-type K-Ras and K-Ras(G12S) (*n* = 3). Data points are plotted as the mean. Error bars represent s.d. and are plotted as dashed lines above and below the data points. Source data

The *KRAS* p.G12S mutation has been observed in thousands of patient tumors^36^, occurring in 2.8% of colorectal adenocarcinoma and 2.5% of nonsmall cell lung cancer^37^. The same mutation on the *HRAS* gene is reported to be an activating mutation^38^ and is a prevalent mutation in Costello syndrome^39^. We asked whether the glycine-to-serine change at codon 12 alone is sufficient to create a mutant K-Ras protein capable of oncogenic signaling. To assess oncogenic transformation by K-Ras(G12S), we took advantage of the Ba/F3 system, an immortalized murine cell line whose growth depends on exogenous interleukin-3 (IL-3) but which exhibits IL-3-independent growth upon transformation. We generated Ba/F3 variants that stably express wild-type K-Ras or K-Ras(G12S) by infecting the cells with ecotropic retroviruses, and verified the ectopic expression of K-Ras by immunoblot using antibodies specific for pan-Ras- and Ras(G12S) (Fig. 1a). We also generated a variant expressing K-Ras(G12C), a common hotspot mutant of K-Ras in human cancer, as a comparator. Relative to the parental line, Ba/F3 cells expressing K-Ras(G12S) and K-Ras(G12C) had elevated phospho-ERK and phospho-AKT levels, whereas those expressing wild-type K-Ras did not (Fig. 1a). Consistent with this observation, cells expressing K-Ras(G12S) and K-Ras(G12C) continued to proliferate at comparable rates after IL-3 had been removed from the culture medium (Fig. 1b). Together, these results suggest that *KRAS* p.G12S is an oncogenic driver mutation with similar transformation potential to *KRAS* p.G12C.

To understand the effects of the G12S mutation on protein structure, we solved a 1.7-Å crystal structure of K-Ras(G12S)•GDP (Fig. 1c), in which K-Ras(G12S) adopts a conformation highly analogous to reported structures of GDP-bound wild-type K-Ras and the oncogenic mutant K-Ras(G12C). In addition, the well-defined density for the P-loop shows that the mutant serine is oriented similarly to the mutant cysteine K-Ras(G12C) (Fig. 1c, insets).

We next asked whether the G12S mutation hampers the rate of GTP hydrolysis, a common biochemical mechanism that confers functional activation and extended pro-growth signaling to K-Ras hotspot mutants^5^. We measured single-turnover GTP hydrolysis by K-Ras(G12S) using a purine nucleoside phosphorylase-coupled assay that monitors free phosphate formation (Fig. 1d). Compared with wild-type K-Ras, K-Ras(G12S) showed a diminished intrinsic GTP hydrolysis rate, and importantly, was insensitive to GTPase-activating protein NF1-mediated acceleration.

### A β-lactone ligand acylates the mutant serine

The residual intrinsic hydrolytic activity of GTP suggests that the GDP-bound state may constitute a substantial population of cellular K-Ras(G12S) and may be targetable by small-molecule ligands, especially those that irreversibly engage the protein through covalent bond formation. To design compounds targeting K-Ras(G12S), we drew lessons from the successful drug discovery efforts directed against K-Ras(G12C)^9,11,15,40^, as well as a family of β-lactone-containing natural products (for example, salinosporamide A, omuralide) that inhibit the 20S proteasome by forming a covalent bond with the catalytic threonine (Thr1)^34,35^. We chose to target the S-IIP because it proves to be a privileged drug-binding pocket for K-Ras and offers direct access to the mutant residues at codon 12.

**Fig. 2:**
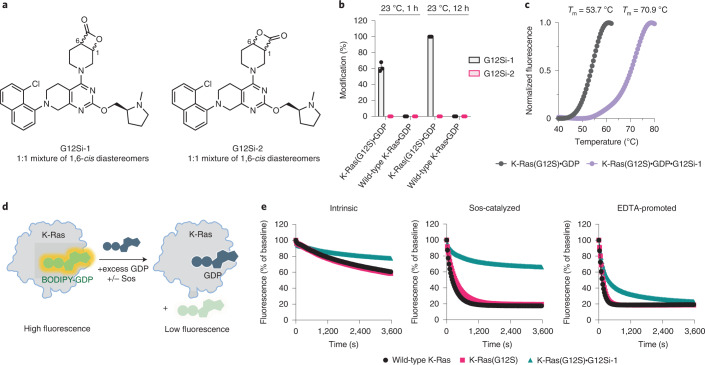
A β-lactone ligand acylates the mutant serine of K-Ras(G12S). **a**, Structures of β-lactones G12Si-1 and G12Si-2. **b**, Covalent modification of 4 µM recombinant K-Ras(G12S)•GDP or wild-type K-Ras•GDP proteins treated with 10 µM G12Si-1 or G12Si-2 assessed by whole-protein MS (*n* = 3). Error bars represent s.d. **c**, Differential scanning fluorimetry of unmodified K-Ras(G12S)•GDP and the K-Ras(G12S)•GDP•G12Si-1 complex (*n* = 3). **d**, Illustration of a biochemical assay that monitors nucleotide exchange using a fluorescent GDP analog. **e**, Nucleotide exchange of wild-type K-Ras, K-Ras(G12S) or K-Ras(G12S)•G12Si-1 adduct in the presence of magnesium (intrinsic), Sos or EDTA (*n* = 3).

**Extended Data Fig. 1:**
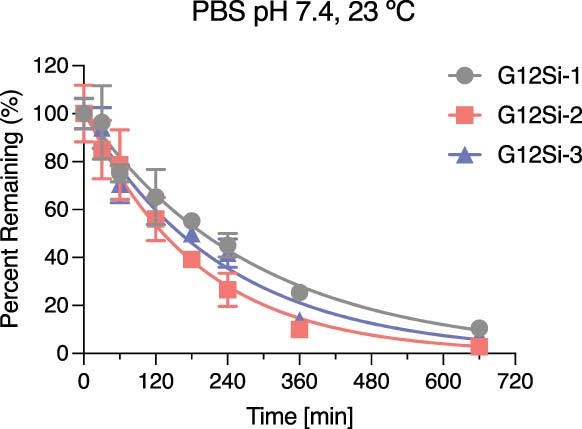
Stability of G12Si-1, G12Si-2 and G12Si-3 in phosphate-buffered saline (PBS) pH 7.4. Data is presented as mean values ± standard deviations (n = 3).

**Extended Data Fig. 2:**
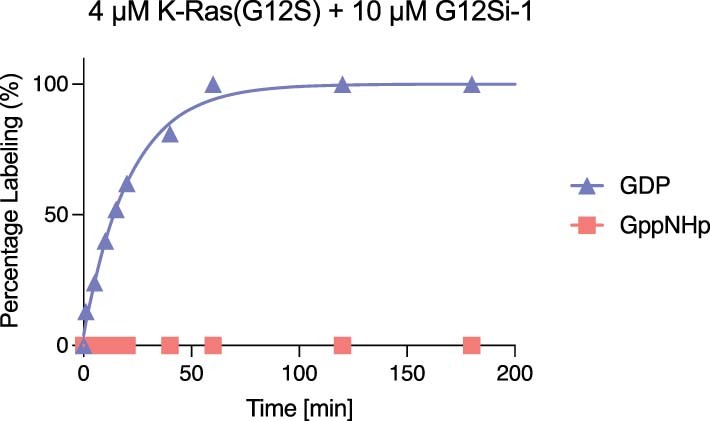
G12Si-1 does not react with GppNHp-loaded K-Ras(G12S). Modification of K-Ras(G12D)•GDP and K-Ras(G12D)•GppNHp by G12Si-1 was monitored by whole protein mass spectrometry.

We synthesized G12Si-1 (**1**) and G12Si-2 (**2**) (Fig. 2a) by attaching a pair of β-lactone electrophiles to the tetrahydropyridopyrimidine moiety found in the clinical candidate MRTX849 and evaluated their ability to covalently engage recombinant K-Ras(G12S) at the mutant serine residue using whole-protein MS. Covalent adduct formation was observed between G12Si-1 (10 µM) and K-Ras(G12S)•GDP (4 µM), with the extent of modification reaching 64% after 1 h at 23 °C and 100% after 12 h (Fig. 2b). By contrast, G12Si-2, a regioisomer of G12Si-1, did not yield any covalent adduct under identical conditions, and neither of these compounds formed covalent adducts with wild-type K-Ras protein. This marked difference in reactivity toward K-Ras(G12S) appeared to be a result of ligand–protein recognition, because G12Si-1 and G12Si-2 showed similar intrinsic reactivity, as measured by their solvolysis rates in PBS (Extended Data Fig. 1). The adduct formation was accompanied by demonstrable thermal stabilization (Fig. 2c), increasing the melting temperature (*T*_m_) of K-Ras(G12S)•GDP from 53.7 °C to 70.9 °C (+17.2 °C). The reaction between G12Si-1 and K-Ras(G12S) is selective for the GDP-bound state; no reaction was observed with guanosine-5′-[β,γ-imido]triphosphate (GppNHp)-loaded K-Ras(G12S) protein under identical conditions (Extended Data Fig. 2).

To assess whether G12Si-1 affects nucleotide cycling of K-Ras, we performed nucleotide-exchange experiments by monitoring the exchange of a fluorescent GDP analog (BODIPY-GDP) for unlabeled GDP in the presence of the guanine nucleotide-exchange factor Son of Sevenless (Sos) or the metal chelator EDTA (Fig. 2d,e). As seen with S-IIP ligands of K-Ras(G12C), G12Si-1 blocked Sos-catalyzed exchange and decreased the rate of EDTA promoted exchange.

**Fig. 3:**
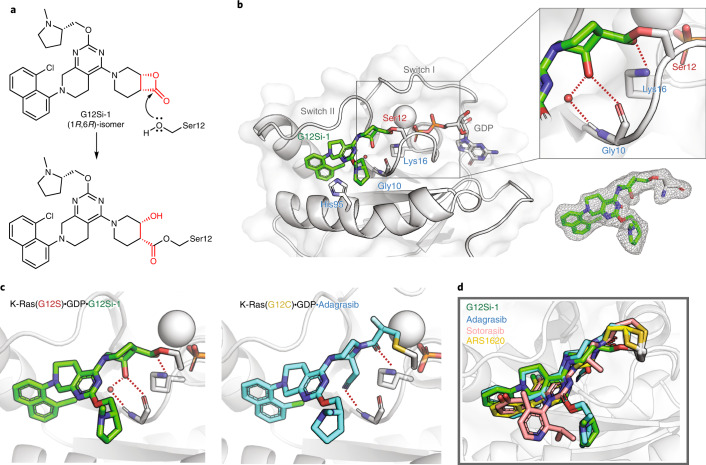
G12Si-1 binds covalently to Ser12 in the switch II pocket of K-Ras(G12S). **a**, Scheme of the nucleophilic ring opening of the β-lactone in G12Si-1 by serine 12. **b**, The X-ray co-crystal structure of the K-Ras(G12S)•GDP•G12Si-1 complex. 2*F*_o_ – *F*_c_ map for the ligand G12Si-1 and serine 12 is depicted in gray mesh (1.0*σ*). **c**, Crystal structure of the K-Ras(G12S)•GDP•G12Si-1 adduct and the K-Ras(G12C)•GDP•adagrasib adduct (PDB: 6USZ). **d**, Superimposition of the conformations of covalent ligands of the S-IIP: G12Si-1 (this work), adagrasib (PDB: 6USZ), sotorasib (PDB: 6OIM), ARS1620 (PDB: 5V9U). For X-ray crystallography data collection and refinement statistics, see Supplementary Table 1.

**Extended Data Fig. 3:**
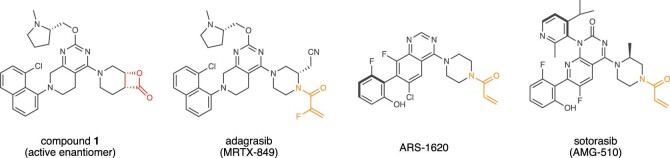
Comparison of the chemical structures of G12Si-1 (this work), adagrasib, ARS1620, and sotorasib. The electrophile portion of each compound is highlighted in red (serine-targeting) or orange (cysteine-targeting).

β-Lactones are highly strained electrophiles with an estimated strain energy of 22.7 kcal mol^−1^ (ref. ^41^). We hypothesized that adduct formation between K-Ras(G12S)•GDP and G12Si-1 involved a nucleophilic attack from the mutant serine residue to the carbonyl group of the β-lactone in G12Si-1 and subsequent ring opening (Fig. 3a). To test this hypothesis, we solved a 2.0-Å crystal structure of the K-Ras(G12S)•GDP•G12Si-1 complex (Fig. 3b). Comparison of this structure with previously reported co-crystal structures of S-IIP ligands with K-Ras(G12C) shows that G12Si-1 binds in the S-IIP and adopts an orientation similar to the G12C ligands (r.m.s. deviation 0.273 Å; Fig. 3c,d and Extended Data Fig. 3). Well-defined electron density confirmed that Ser12 was acylated by G12Si-1 (Fig. 3b, inset), giving rise to a protein–drug complex mediated by an ester linkage in which the strained β-lactone ester is replaced by an unstrained ester. We also observed that the carbonyl oxygen of this ester group is engaged in a hydrogen bond with Lys16, and that the secondary alcohol resulting from the β-lactone opening formed a hydrogen bond with the backbone carbonyl of glycine 10 (Fig. 3b, inset). A water molecule was also observed bridging the same secondary alcohol and the backbone N-H of glycine 10. Hydrogen bonding between Lys16 and the carbonyl group on acrylamide electrophiles has been proposed to greatly enhance the reactivity for K-Ras(G12C) inhibitors^42^. These anchoring interactions explain the drastic difference in activity between the closely related compounds G12Si-1 and G12Si-2, because the latter not only has a misaligned electrophile, but also lacks the correct geometry to form either of these hydrogen bonds.

### Optimized β-lactone ligands suppress K-Ras(G12S) signaling

**Fig. 4:**
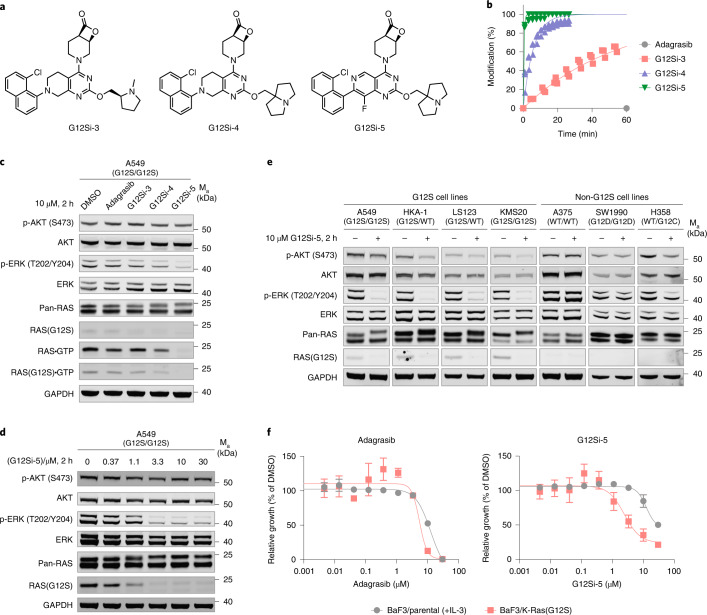
Optimized β-lactone ligands suppress K-Ras(G12S) signaling in cells. **a**, Structures of K-Ras(G12S) ligands G12Si-3, G12Si-4 and G12Si-5. **b**, Time-dependent covalent modification of recombinant K-Ras(G12S)•GDP protein by 10 µM compound at 23 °C assessed by whole-protein MS (*n* = 3, replicates are plotted as individual data points). **c**, Immunoblot of A549 cells treated with 10 µM adagrasib, G12Si-3, G12Si-4 or G12Si-5 for 2 h. Data are representative of two independent experiments. **d**, Immunoblot of A549 cells treated with various concentrations of G12Si-5 for 2 h. Data are representative of two independent experiments. **e**, Immunoblot of A549, A375, SW1990 and H358 cells treated with DMSO or 10 µM G12Si-5 for 2 h. Data are representative of two independent experiments. WT, wild-type. **f**, Relative growth of Ba/F3 parental cells (+10 ng ml^−1^ IL-3) and Ba/F3:K-Ras(G12S) cells (no IL-3) after treatment with adagrasib or G12Si-5 for 72 h. Data are presented as mean ± s.d. (*n* = 3) and are representative of four independent experiments. Source data

**Extended Data Fig. 4:**
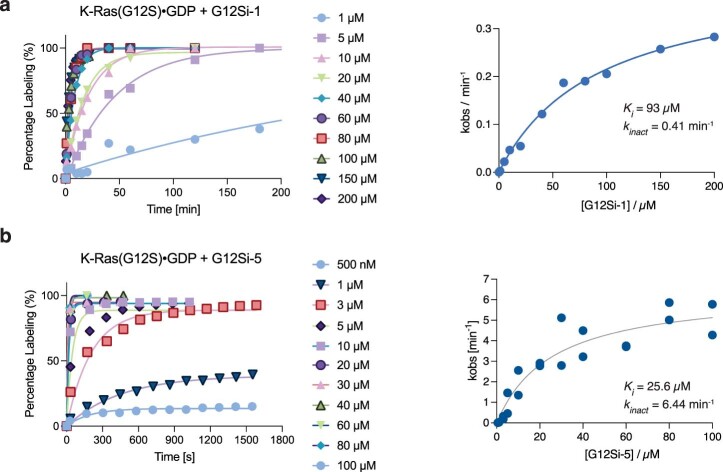
Measurement of kinetic constants for G12Si-1 and G12Si-5. Measurement of *K*_*i*_ and *k*_*inact*_ for the reactions a) between K-Ras(G12S)•GDP and G12Si-1, b) between K-Ras(G12S)•GDP and G12Si-5. For each kinetic curve, *k*_*obs*_ was determined by fitting the data to a first-order kinetic model. Replicates are plotted as individual data points in the right panel. See Methods section for additional details on data analysis.

**Extended Data Fig. 5:**
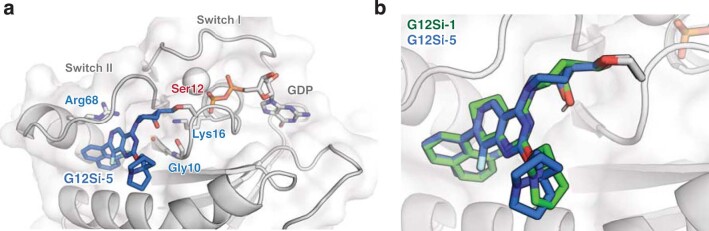
Co-crystal structure of the K-Ras(G12S)•GDP•G12Si-5 adduct. **a**) Co-crystal structure of the K-Ras(G12S)•GDP•G12Si-5 adduct. **b**) Superimposed conformations of G12Si-1 and G12Si-5 in their respective co-crystal structures with K-Ras(G12S)•GDP. For X-ray crystallography data collection and refinement statistics, see Supplementary Table 1.

This structural analysis also reaffirmed the β-lactone, being part of a [4.2.0] bicyclic system, as the core pharmacophore in G12Si-1. Although G12Si-1 was prepared as a mixture of diastereomers, it is evident that the (1*R*,6*R*)-isomer (depicted in Fig. 3a) was the dominantly active diastereomer. Because we sought to improve the potency of G12Si-1, we first synthesized the (1*R*,6*R*)-isomer of G12Si-1 with high diastereomeric purity (**3**, hereafter referred to as G12Si-3). Because the reversible inhibitory constant (*K*_i_, 97 µM) and the first-order rate constant (*k*_inact_, 0.41 min^−1^) that we measured for G12Si-1 (Extended Data Fig. 4a) suggested potential benefits of improving the reversible binding, we also varied the *N*-methylprolinol substituent and the tetrahydropyridopyrimidine moiety based on recent patent literature^43^, yielding G12Si-4 (**4**) and G12Si-5 (**5**) (Fig. 4a). G12Si-3, G12Si-4 and G12Si-5 all underwent enhanced reaction with recombinant K-Ras(G12S)•GDP protein, with G12Si-5 being the most potent, reaching 100% modification in <10 min at 10 µM (Fig. 4b). By comparison, the K-Ras(G12C)-targeting clinical candidate adagrasib, bearing a 2-fluoroacrylamide electrophile, did not react with K-Ras(G12S)•GDP even after extended incubation. Compared with G12Si-1, G12Si-5 exhibited both improved reversible binding affinity (*K*_i_ = 26 µM) and an accelerated reaction (*k*_inact_ = 6.4 min^−1^) with K-Ras(G12S) (Extended Data Fig. 4b). A co-crystal structure of G12Si-5 and K-Ras(G12S)•GDP (Extended Data Fig. 5) revealed that G12Si-5 binds in the S-IIP with a similar pose to that observed for G12Si-1, but with its piperidine ring adopting a chair conformation rather than the twisted-boat seen in K-Ras(G12S)•G12Si-1 adduct.

**Extended Data Fig. 6:**
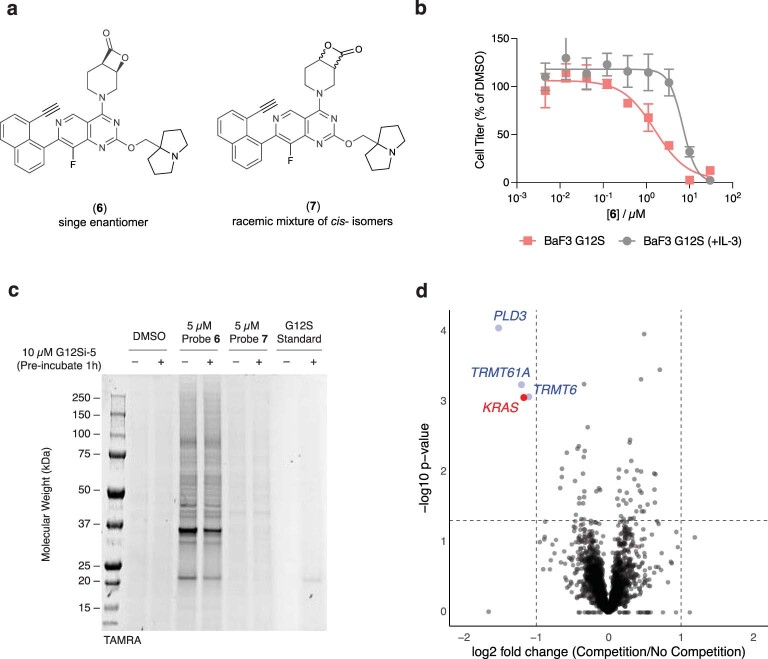
Analysis of the cellular targets of G12Si-5 using an alkyne analog. **a**) Structures of probes **6** (an analog of G12Si-5) and **7** (regioisomer of **6**). **b**) Relative growth of Ba/F3:K-Ras(G12S) cells in the presence (10 ng/mL) or absence of IL-3 after treatment with **6** for 72 h. Data is presented as mean values ± standard deviations (*n* = 3). **c**) In-gel fluorescence image of SDS-PAGE-resolved lysates of compound-treated A549 cells after click-conjugation of the tetramethylrhodamine (TAMRA) fluorophore. Data is representative of three independent experiments. **d**) Volcano plot of proteins captured by neutravidin from A549 cells treated with **6** (5 µM) after click conjugation of biotin. These cells were pre-treated with G12Si-5 (10 µM, “Competition”) or DMSO (“No Competition”) for 1 h prior to the addition of probe **6**. Proteins were quantified using tandem mass tags. Fold change is calculated as ratio of mean intensities (three biological replicates per condition). *p*-values are calculated using unpaired, two-tailed Student’s *t*-test. See Methods section for mass spectrometry methods and Supplementary Dataset 1 for a full list of identified proteins. Source data

We asked whether these optimized β-lactones allow targeted inhibition of K-Ras(G12S) in genetically characterized cancer cell lines. We treated A549 cells (homozygous *KRAS* p.G12S mutation) with 10 µM adagrasib, G12Si-3, G12Si-4 or G12Si-5 for 2 h and monitored phospho-AKT and phospho-ERK levels. We also took advantage of a mutant selective antibody that recognizes Ras(G12S) and does not react with covalently modified K-Ras(G12S) protein (see Supplementary Figs. 2 and 3 for its validation) and used it in concert with Raf-RBD pulldown to assess the intracellular level of GTP-bound K-Ras(G12S). Although neither adagrasib nor G12Si-3 had any observable effects on these markers, treatment by G12Si-4 decreased the level of Ras•GTP, and treatment by G12Si-5 led to nearly complete loss of Ras•GTP and concomitant inhibition of phospho-ERK (Fig. 4c). These changes were accompanied by direct adduct formation with cellular K-Ras(G12S), as seen in the upward shift of the K-Ras band in the anti-Ras blot and the disappearance of the signal in the anti-Ras(G12S) blot. Inhibition of Ras signaling by G12Si-5 in A549 cells was dose-dependent, with an apparent half-maximum inhibitory concentration (IC_50_) of approximately 3 µM under these treatment conditions (Fig. 4d). A terminal alkyne-containing analog of G12Si-5 (Probe **6**; Extended Data Fig. 6a,b) enabled us to use click chemistry to enrich the covalently modified proteins in A549 cells and analyze them using LC–MS/MS. Using competition proteomics, we found K-Ras(G12S) to be a selective target of G12Si-5 with high stoichiometric engagement (Extended Data Fig. 6c,d and Supplementary Dataset 1). We also observed high stoichiometric engagement for three other proteins (PLD3, TRMT61A and TRMT6), which merit future investigation because they could represent targets that are either specific to this chemical scaffold or common for this class of electrophile, similar to the general reactivity of FAM213A and AHR with clinically approved cysteine-targeting acrylamide electrophiles^44^.

We next examined four cell lines with confirmed *KRAS* p.G12S mutations (see Supplementary Fig. 1 for sequencing data). In each of these cell lines, treatment with G12Si-5 led to a reduction in the phospho-ERK level and gel-mobility shift of the K-Ras protein (Fig. 4e). Meanwhile, G12Si-5 did not perturb the signaling in A375 cells (wild-type KRAS) or SW1990 cells (homozygous *KRAS* p.G12D), and showed only weak inhibition of phospho-Erk in H358 cells (heterozygous *KRAS* p.G12C), an effect we ascribed to possible ring opening of the β-lactone by the nucleophilic cysteine.

**Extended Data Fig. 7:**
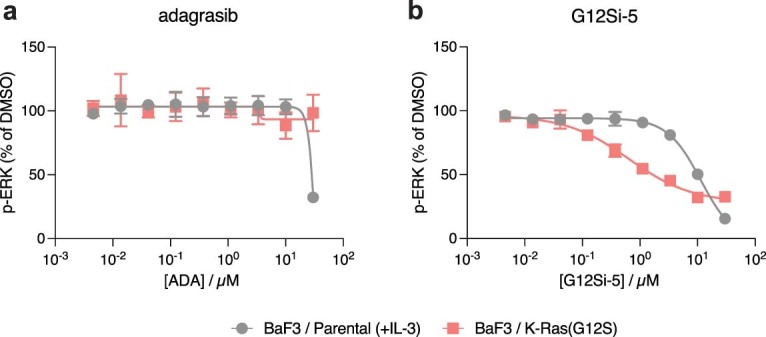
G12Si-5 inhibits phospho-ERK signaling in BaF3/K-Ras(G12S) cells. Phospho-ERK levels of BaF3 parental cells (+10 ng/mL IL-3) and BaF3/K-Ras(G12S) cells (no IL-3) after treatment with (**a**) adagrasib or (**b**) G12Si-5 for 1 h. Data is presented as mean values ± standard deviations (*n* = 3).

**Extended Data Fig. 8:**
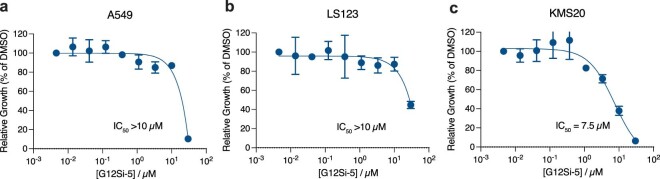
Growth inhibition of KRAS *p*. G12S mutant cell lines by G12Si-5. Relative growth of (**a**) A549, (**b**) LS123 and (**c**) KMS20 cells after treatment with G12Si-5 for 72 h. Data is presented as mean values ± standard deviations (*n* = 3).

Although these data confirmed the specificity of G12Si-5 against the *KRAS* G12S allele, we reasoned that the chemoselectivity encoded by the β-lactone group could afford a therapeutic window between cells expressing the mutant K-Ras(G12S) and those expressing wild-type K-Ras. To test this, we compared the effects of G12Si-5 on the proliferation of Ba/F3:K-Ras(G12S) cells (without IL-3) and parental Ba/F3 cells (with IL-3). G12Si-5 preferentially inhibited the growth of the K-Ras(G12S)-transduced cells with an IC_50_ of 2.4 µM and, to a lesser extent, that of the parental cells with an IC_50_ of 12.5 µM (Fig. 4f). Notably, the non-G12S-targeting compound adagrasib did not display such selectivity and caused complete cell death at 10 µM for both cell lines. Consistent with this result, treatment of Ba/F3:K-Ras(G12S) cells with compound G12Si-5 led to a dose-dependent reduction in phospho-ERK levels (Extended Data Fig. 7a,b). G12Si-5 also inhibited the growth of KMS20, a patient-derived myeloma cell line with a homozygous *KRAS* p.G12S mutation, with an IC_50_ of 7.5 µM (Extended Data Fig. 8a–c). We did not observe growth inhibition of A549 or LS123 cells below the toxic threshold of 10 µM, two cell lines reported to have low *KRAS* dependency^45–47^ (Extended Data Fig. 8). Although the fivefold selectivity observed with G12Si-5 merits extensive optimization, our data provide the first example of selective targeting of the K-Ras(G12S) mutant using small-molecule agents.

## Discussion

The discovery that the mutant cysteine in K-Ras(G12C) can be exploited by small-molecule electrophiles has fueled renewed efforts to develop agents targeting K-Ras, culminating in the recent U.S. Food and Drug Administration approval of sotorasib with five additional drug candidates under clinical investigation. Nevertheless, therapeutic strategies based on covalent targeting of other hotspot mutants of K-Ras have not been reported. Compared with cysteine, the acquired serine residue in K-Ras(G12S) has much weaker nucleophilicity and does not react with electrophiles tailored for K-Ras(G12C). Inspired by a family of threonine-targeting natural products containing the four-membered strained β-lactone, we sought to target the G12S allele of K-Ras. Using structure-guided chemical design, we identified β-lactone as a privileged electrophile for the acquired serine in K-Ras(G12S) and synthesized S-IIP ligands that rapidly undergo covalent engagement with this mutant residue. Similar to the case of K-Ras(G12C) inhibitors, even though our compounds do not bind to the GTP-bound form of K-Ras(G12S), its intrinsic GTPase activity is sufficient to support the complete irreversible trapping of this mutant allele and allele-specific suppression of oncogenic cellular signaling.

Natural and synthetic β-lactones^48^ are known to undergo ring opening following nucleophilic attack from catalytically active serine or threonine residues in enzymes (for example, omuralide/20S proteasome, lipstatin/pancreatic lipase and palmostatin B/acyl protein thioesterase). Yet, to the best of our knowledge, such reactivity has not been observed with noncatalytic serines, including the acquired serines in mutant proteins. Our work demonstrates that chemical acylation of a noncatalytic serine can be achieved using β-lactone electrophiles^49,50^. Although we focused on K-Ras(G12S) in this study, we are optimistic that our strategy adds to the toolbox of serine-targeting electrophiles and may be adopted for other targets with ligand-accessible serine residues.

## Methods

### Safety notes

All experiments were performed using standard personal protective equipment. All chemical syntheses were performed in ventilated fume hoods operating at a face velocity >90 f.p.m. Handling of Biosafety Level 2 materials was performed according to UCSF Office of Environment, Health and Safety standards. No unexpected or unusually high safety hazards were encountered.

### Cell culture

Ba/F3 cells were a gift from T. Bivona (UCSF) and were maintained in RPMI 1640 (Gibco, catalog no. 11875093) supplemented with 10% heat-inactivated FBS (Axenia Biologix) and 10 ng ml^−1^ recombinant mouse IL-3 (Gibco, catalog no. PMC0031). A549 cells were obtained from UCSF Cell Culture Facility and maintained in high-glucose (4.5 g l^−1^) DMEM (Gibco, catalog no. 11995073) supplemented with 4 mM l-glutamine, 1 mM sodium pyruvate and 10% heat-inactivated FBS (Axenia Biologix). LS123 cells were obtained from ATCC (CCL-255) and maintained in high-glucose (4.5 g l^−1^) DMEM (Gibco, catalog no. 11995073) supplemented with 4 mM l-glutamine, 1 mM sodium pyruvate and 10% heat-inactivated FBS (Axenia Biologix). HKA-1 cells were obtained from JRCB Cell Bank (JRCB1017) and maintained in high-glucose (4.5 g l^−1^) DMEM (Gibco, catalog no. 11995073) supplemented with 4 mM l-glutamine, 1 mM sodium pyruvate and 10% heat-inactivated FBS (Axenia Biologix). KMS20 cells were from JRCB Cell Bank (JRCB1196) and maintained in RPMI 1640 (Gibco, catalog no. 11875093) supplemented with 10% heat-inactivated FBS (Axenia Biologix). NCI-H358 cells were obtained from ATCC (CRL-5807) and maintained in high-glucose (4.5 g l^−1^) DMEM (Gibco, catalog no. 11995073) supplemented with 4 mM l-glutamine, 1 mM sodium pyruvate and 10% heat-inactivated FBS (Axenia Biologix). A375 cells were obtained from ATCC (CRL-1619) and maintained in high-glucose (4.5 g l^−1^) DMEM (Gibco, catalog no. 11995073) supplemented with 4 mM l-glutamine, 1 mM sodium pyruvate and 10% heat-inactivated FBS (Axenia Biologix). SW1990 cells were obtained from ATCC (CRL-2172) and maintained in high-glucose (4.5 g l^−1^) DMEM (Gibco, catalog no. 11995073) supplemented with 4 mM l-glutamine, 1 mM sodium pyruvate and 10% heat-inactivated FBS (Axenia Biologix).

Cells were passed for at least two generations after cryorecovery before they were used in the assays. All cell lines tested mycoplasma negative using MycoAlert Mycoplasma Detection Kit (Lonza). When indicated, cells were treated with drugs at 40%–60% confluency at a final DMSO concentration of 1%. At the end of the treatment period, cells were placed on ice. Unless otherwise indicated, adherent cells were washed once with ice-cold PBS (1 ml), scraped with a spatula and pelleted by centrifugation (500*g*, 5 min). Suspension cells were pelleted by centrifugation (500*g*, 5 min), washed with 1 ml of ice-cold PBS and pelleted again. Cells were lysed in RIPA buffer supplemented with protease and phosphatase inhibitors (cOmplete and phosSTOP, Roche) on ice for 10 min. For RBD pulldown experiments, cells were lysed in coimmunoprecipitation (Co-IP) lysis buffer in lieu of RIPA buffer. Lysates were clarified by high-speed centrifugation (19,000*g*, 10 min). Lysate concentrations were determined with a BCA protein assay (Thermo Fisher Scientific) and adjusted to 2 mg ml^−1^ with additional RIPA buffer (or Co-IP lysis buffer). Samples were mixed with 5× SDS loading dye and heated at 95 °C for 5 min.

### Analysis of GTP-bound Ras by Raf-RBD pulldown

Ras•GTP pulldown was performed with glutathione S-transferase-tagged Ras binding domain (GST-RBD) immobilized on agarose beads (cytoskeleton, 10 µl, 30 µg of loaded GST-RBD). Lysate (50 µl, 2 mg ml^−1^) was mixed with 10 µl of beads and the suspension was incubated at 4 °C for 1 h with constant end-to-end rotation. The beads were settled by centrifugation (500*g*, 5 min) and the supernatant was carefully removed. The beads were washed with 2× 500 µl of ice-cold Co-IP wash buffer. Bound protein was eluted with 10 µl of 2× SDS loading buffer.

### Gel electrophoresis and immunoblot

Unless otherwise noted, SDS–PAGE was run with Novex 12% Bis-Tris gel (Invitrogen) in MES running buffer (Invitrogen) at 200 V for 60 min following the manufacturer’s instructions. Protein bands were transferred to 0.2-µm nitrocellulose membranes (Bio-Rad) using wet-tank transfer apparatus (Bio-Rad Criterion Blotter) in 1× TOWBIN buffer with 10% methanol at 75 V for 45 min. Membranes were blocked in 5% Bovine serum albumin (BSA) in Tris-buffered saline supplemented with Tween-20 (20 mM Tris, 150 mM NaCl, 0.1% Tween-20, pH 7.6, hereafter referred to as TBST) for 1 h at 23 °C. Primary antibody binding was performed with the indicated antibodies diluted in 5% BSA–TBST at 4 °C for at least 16 h. After washing the membrane three times with TBST (5 min each wash), secondary antibodies (goat anti-rabbit IgG-IRDye 800, 1:5,000, and goat anti-mouse IgG-IRDye 680, 1:5,000, Li-COR) were added as solutions in 5% skim milk–TBST at the dilutions recommended by the manufacturer. Secondary antibody binding was allowed to proceed for 1 h at 23 °C. The membrane was washed three times with TBST (5 min each wash) and imaged on a Li-COR Odyssey fluorescence imager.

### Preparation of mouse stem cell virus (MSCV)

pMSCV-Puro plasmids containing full-length human *KRAS* genes (wild-type, G12S, G12C) were constructed using standard molecule biology techniques by inserting the *KRAS* gene fragment between the BamHI and XhoI sites. Transfection-grade plasmids were prepared using ZymoPure II Plasmid Midiprep kit. EcoPack 293 cells (Takara Bio) were plated in six-well plates (3 × 10^5^ per ml, 2 ml). The next day, cells were transfected with 2.5 µg of pMSCV plasmid using Lipofectamine 3000 following the manufacturer’s instructions. The cells were incubated for 66 h, and the virus-containing supernatants were then collected and passed through a 0.22-µm syringe filter. The harvested virus was used immediately for spinfection of Ba/F3 cells or stored at –80 °C.

### Generation of stable Ba/F3 transductants

One milliliter of MSCV-containing supernatant (vide supra) was added to one well of a six-well plate containing 1 × 10^6^ Ba/F3 cells in 1 ml of media comprised of 60% RMPI 1640, 40% heat-inactivated FBS, 10 ng of mouse IL-3 and 4 µg of polybrene. Cells were spinfected by centrifugation at 2,000*g* for 90 min at room temperature and then placed in the incubator for 24 h. After 1 d, cells were diluted into 10 ml culture medium (RPMI 1640 + 10% heat-inactivated FBS, 10 ng ml^−1^ mouse IL-3) and recovered for a second day after spinfection. On the third day after spinfection, cells were pelleted at 500*g* for 5 min and resuspended in 10 ml of selection medium (RPMI 1640 + 10% heat-inactivated FBS, 10 ng ml^−1^ mouse IL-3, 1.25 µg ml^−1^ puromycin). Cells were maintained under puromycin selection for 4–7 d, splitting as required to maintain a density of <2 × 10^6^ cells per ml. After 7 d, cells were pelleted, washed once with culture medium free of IL-3 (RPMI 1640 + 10% heat-inactivated FBS) and pelleted again before resuspending at 2–4 × 10^5^ cells per ml in culture medium free of IL-3. Cells were maintained under these conditions for 7 d, passaging as needed to maintain a density of <2 × 10^6^ cells per ml. Growth was monitored (Countess II Cell Counter) over these 7 d to confirm that an IL-3-independent population has been achieved.

### Stability of β-lactone compounds in PBS

A 10 µM solution of G12Si-1, G12Si-2 or G12Si-3 in PBS, pH 7.4 was prepared by diluting 1 µl of a 10 mM DMSO stock solution into 999 µl of PBS pH 7.4. At specified time points, samples were taken, and the amount of nonhydrolyzed compound was analyzed by multiple-reaction monitoring on a Waters Xevo G2-XS Quadrupole-TOF system equipped with an Acquity UPLC BEH C18 1.7 µm column, monitoring the transition *m*/*z* 267.62 → 393.15.

### Phospho-ERK assay

Ba/F3 parental cells were pelleted and resuspend in growth medium supplemented with IL-3 (RPMI 1640, 10% heat-inactivated FBS, 10 ng ml^−1^ mouse IL-3 medium) to 1 × 10^7^ per ml. Ba/F3 K-Ras(G12S) transductant cells were pelleted and resuspended in growth medium free of IL-3 (RPMI 1640, 10% heat-inactivated FBS) to 1 × 10^7^per ml. Cells (25 µl) were added to each well of a 96-well plate (2.5 × 10^5^ cells per well), followed by 5 µl of 6× compound solutions (6% DMSO). The mixture was incubated at 37 °C for 1 h. Cell lysis and the subsequent high-throughput phospho-ERK assay was performed using a Cisbio Advanced phospho-ERK (Thr202/Tyr204) cellular kit (PerkinElmer, catalog no. 64AERPEG) following the manufacturer’s instructions.

### GTPase assay

A GTPase assay was performed using an EnzCheck Phosphate Assay Kit (Invitrogen E6646) following a previously reported procedure^5^ with modifications. K-Ras proteins were loaded with GTP as follows. K-Ras proteins were diluted in EDTA buffer (25 mM HEPES 7.5, 150 mM NaCl, 5 mM EDTA, 1 mM DTT) supplemented with 1 mM GTP at 0 °C to 100 µM. After incubation for 1 h on ice, the protein solutions were exchanged into reaction buffer (20 mM HEPES 7.5, 150 mM NaCl, 1 mM DTT) using a PD-10 column following the manufacturer’s instructions. The eluted protein (3.5 ml) was concentrated using a 10K-MWCO Amicon-4 concentrator and protein concentrations were adjusted to 100 µM with reaction buffer. The GTPase assay was performed in a clear 96-well half-volume UV-star plate (Greiner Bio-one, catalog no. 675801) as follows. To each well was added the following components: 50 µl of protein at 100 µM, 20 µl of 2-amino-6-mercapto-7-methylpurine riboside at 1.0 mM, 5 µl of purine nucleotide phosphorylase at 0.1 U µl^−1^. Control conditions in which the protein solution was substituted for reaction buffer (blank control) or 100 µM free phosphate standard (Pi control) were also included. The GTPase reaction was initiated by the addition of 25 µl of 4× Mg buffer (40 mM MgCl_2_) or 4× NF1 buffer (200 µM NF1-GRD, 40 mM MgCl_2_). This should be completed within 15 s. The absorbance at 360 nm was immediately read every 30 s for 3,600 s at 23 °C using a TECAN Spark 20 M plate reader. For each data point, absorbance was subtracted from the reading in the blank control, then normalized to the difference between Pi control and blank control, and reported as percentage of theoretical maximum hydrolysis.

### Sos- or EDTA-mediated nucleotide-exchange assay

This assay was performed as previously reported^15^ with slight modifications. BODIPY-GDP-loaded K-Ras proteins were prepared freshly as follows. To a 10 µM solution of K-Ras(wild-type)•GDP, K-Ras(G12S)•GDP or K-Ras(G12S)•GDP•G12Si-1 in size exclusion chromatography (SEC) buffer (1 ml) was added sequentially BODIPY-GDP (5 mM, 40 µl, final concentration 200 µM; Thermo Fisher Scientific) and Na-EDTA pH 8.0 (0.5 M, 5 µl, final concentration 2.5 mM). The mixture was incubated at 23 °C for 1 h, and a solution of MgCl_2_ (1.0 M, 20 µl, final concentration 10 mM) was added. The protein solution was run through a PD-10 column to remove excess nucleotide following the manufacturer’s protocol. Briefly, sample (~1.0 ml) and excess buffer (1.5 ml) were loaded onto the column (equilibrated with NucEx buffer), and desalted protein was eluted with NucEx buffer (3.5 ml). Protein concentration was measured using a Bradford assay and adjusted to 1.25 µM with NucEx buffer. Twelve microliters of this solution (in triplicate for each condition) was added to wells of a black 384-well low-volume assay plate (Corning 4514). Three microliters of either 1 mM GDP, 1 mM GDP + 5 µM Sos or 1 mM GDP + 40 mM EDTA (all prepared in NucEx buffer) was added rapidly to the wells using a multichannel pipette. This should take less than 15 s to finish. The plate was immediately placed in a TECAN Spark 20M plate reader, and fluorescence for BODIPY (excitation 488 nm, emission 520 nm) was read every 30 s over 1 h. Fluorescence intensity was normalized to values at time 0 and plotted against time. The observed rate constant (*k*_obs_) was derived by fitting the curve to first-order kinetic equation

$$F = (F_0 - F_\infty ){{{\mathrm{exp}}}}\left[ { - k_{\mathrm{obs}}t} \right]{{{\mathrm{ }}}} + {{{\mathrm{ }}}}F_\infty$$

and plotted against time.

### Differential scanning fluorimetry

The protein of interest was diluted with SEC buffer (20 mM HEPES 7.5, 150 mM NaCl, 1 mM MgCl_2_) to 8 µM. This solution was dispensed into the wells of a white 96-well PCR plate in triplicate (25 µl per well). Fluorescence was measured at temperature intervals of 0.5 °C intervals every 30 s from 25 °C to 95 °C on a Bio-Rad CFX96 quantitative PCR system using the FRET setting. Each dataset was normalized to the highest fluorescence and the normalized fluorescence reading was plotted against temperature in GraphPad Prism v.9.0. *T*_m_ values were determined as the temperature(s) corresponding to the maximum(ma) of the first derivative of the curve.

### Detection of covalent modification of K-Ras proteins by mass spectrometry

Test compounds were prepared as 100× stock solutions in DMSO. K-Ras proteins were diluted with SEC buffer (20 mM HEPES 7.5, 150 mM NaCl, 1 mM MgCl_2_) to 400 nM or 1 µM. In a typical reaction, 0.5 µl of 100× compound stock was mixed with 50 µl of diluted K-Ras protein, and the resulting mixture was incubated for the desired length of time. The extent of modification was assessed by electrospray MS using a Waters Xevo G2-XS system equipped with an Acquity UPLC BEH C4 1.7 µm column. The mobile phase was a linear gradient of 5%–95% acetonitrile/water + 0.05% formic acid. For kinetic measurements, a 2× compound solution was first prepared in SEC buffer, which was then mixed with 400 nM K-Ras(G12S) protein at 1:1 (v/v) ratio. Injection time stamps were used to calculate elapsed time.

### Cell viability assay

Cells were seeded into 96-well white flat-bottom plates (1,000 cells per well) (Corning) and incubated overnight. Cells were treated with the indicated compounds in a nine-point threefold dilution series (100 μl final volume) and incubated for 72 h. Cell viability was assessed using a commercial CellTiter-Glo (CTG) luminescence-based assay (Promega). Briefly, the 96-well plates were equilibrated to room temperature before the addition of diluted CTG reagent (100 μl) (1:4 CTG reagent/PBS). Plates were placed on an orbital shaker for 30 min before recording luminescence using a Spark 20 M (Tecan) plate reader.

### In-gel fluorescence

One day before treatment, A549 cells were plated in six-well tissue culture plates (3 × 10^5^ cells per well). The next day, cells were pretreated with DMSO or G12Si-5 (10 µM, 0.1% final DMSO) at 37 °C for 1 h. Probes (DMSO, **6** or **7**) were then added to the culture media as a 1,000× DMSO solution. The cells were incubated for an additional 1 h at 37 °C. At the end of the treatment period, cells were placed on ice and the media were removed by aspiration. Cells were washed once with ice-cold PBS (1 ml), scraped with a spatula and pelleted by centrifugation (500*g*, 5 min). Cells were lysed in Co-IP lysis buffer supplemented with protease and phosphatase inhibitors (cOmplete and phosSTOP, Roche) on ice for 10 min. Lysates were clarified by high-speed centrifugation (19,000*g*, 10 min). Lysate concentrations were determined using a BCA protein assay (Thermo Fisher Scientific) and adjusted to 2 mg ml^−1^ with additional Co-IP lysis buffer. For each treatment condition, 20 µl of lysate was mixed with 5 µl of freshly prepared 5× TAMRA-N_3_ Click Master Mix. The reactions were allowed to proceed in the dark for 1.5 h, then 5 µl of 6× SDS loading buffer was added to the reaction mixture. Fifteen microliters of the resulting mixture was resolved by SDS–PAGE on a Novex 4%–12% Bis-Tris gel (Invitrogen) in MES running buffer (Invitrogen) at 200 V for 60 min. The gel was washed successively with gel-fixation buffer (40% methanol, 10% acetic acid, 2 × 1 h), 50% methanol (1 h) and water (1 h), then scanned on a Typhoon FLA 9000 scanner (532 nm, LPG filter).

### Liquid chromatography tandem mass spectrometry analysis of proteins covalently modified by Probe 6

One day before treatment, A549 cells were added to 15-cm tissue culture dishes (4 × 10^6^ cells per dish). The next day, cells were treated with 10 µM G12Si-5 (competition) or DMSO (no competition) for 1 h. Probe **6** was then added to the culture media and cells were incubated for an additional 1 h. Each treatment condition was performed in biological duplicate. Cells were then placed on ice, and the media removed by aspiration. Cells were washed once with ice-cold PBS (10 ml), scraped with a spatula and pelleted by centrifugation (500*g*, 5 min). Cells were lysed in Co-IP lysis buffer supplemented with protease and phosphatase inhibitors (cOmplete and phosSTOP; Roche) on ice for 10 min. Lysates were clarified by high-speed centrifugation (19,000*g*, 10 min). Concentrations of lysates were determined using a BCA protein assay (Thermo Fisher Scientific) and adjusted to 2 mg ml^−1^ with additional Co-IP lysis buffer. Lysate (500 µl) was mixed with 80 µl of high-capacity neutravidin agarose beads (Pierce, 50% v/v suspension, washed with Co-IP lysis buffer), and the mixture was incubated overnight at 4 °C with constant end-to-end rotation to deplete the endogenously biotinylated proteins. The resin was removed by filtration and the lysate was mixed with 125 µl of freshly prepared 5× Biotin-N_3_ Click Master Mix. The reaction was allowed to proceed at 23 °C for 1.5 h. Then, 80 µl of high-capacity neutravidin agarose beads (Pierce; 50% v/v suspension, washed with Co-IP lysis buffer) were added, and the mixture was incubated at 4 °C for 1 h with constant end-to-end rotation. The beads were washed successively with:

1. PBS + 1% NP-40 + 0.1% SDS, three times, 23 °C
2. Freshly prepared 6 M urea in PBS, three times, 4 °C
3. Ice-cold PBS, three times, 4 °C
4. Digestion buffer (20 mM HEPES, 2 mM CaCl_2_, pH 8.0), twice, 4 °C.

Each wash was performed by spinning down the resin (1,500*g*, 5 min), removing the supernatant, resuspending the resin in 0.5 ml of wash buffer, and rotating the resin–buffer mixture for 10 min at the specified temperature. Beads were moved to new tubes after the first, fourth and seventh washes.

After the washes, beads were resuspended in 80 µl of digestion buffer and mixed with 2 µl of 200 mM DTT. The mixture was heated at 56 °C for 30 min. After cooling to 23 °C, 4 µl of 200 mM iodoacetamide was added. After 15 min at 23 °C, 2.1 µl of 200 mM DTT was added. After a further 5 min, 500 ng of trypsin was added to the mixture, and the samples were incubated at 37 °C overnight with constant agitation (1,000 r.p.m., orbital shaker). The tryptic peptides were labeled with TMTsixplex Isobaric Label Reagent Set (Thermo Fisher Scientific, catalog no. 90061) following the manufacturer’s instructions. After completion of the labeling reaction and quenching with 0.5% hydroxylamine, the peptide mixtures were pooled in a single tube, and 60 µl of 10% formic acid was added to acidify the mixture. The acidified mixture was dried by vacuum centrifugation (Genevac). The residue, which contains labeled peptides, salts and quenched TMT reagents, was resuspended in 500 µl of 5% acetonitrile/water + 0.1% formic acid. The sample was loaded onto a Waters Oasis HLB column (Waters, catalog no. 186000383; 1 ml, 30 mg sorbent), the column was washed with 0.1% formic acid (3 × 1 ml), and the peptides were eluted with 70% acetonitrile/water + 0.1% formic acid (2 × 200 µl). The eluted peptides were dried under vacuum (Genevac). The dried peptides were fractionated using a Pierce High pH Reverse-Phase Peptide Fractionation Kit (Thermo Fisher Scientific, catalog no. 84868) following the manufacturer’s instructions. Peptide fractions were dried individually and resuspended in 100 µl of 0.1% formic acid. Peptides were resolved on an Easy-Spray nano-HPLC column (Thermo Fisher Scientific, catalog no. ES800A; 150 mm length, 3 µl particle size, 100-Å particle size) over a 180-min gradient of 2%–37% acetonitrile/water + 0.1% formic acid and analyzed by a Orbitrap Eclipse tribrid mass spectrometer (precursor range: 400–1,600 *m*/*z*, charge state 2–6, MS1 Orbitrap resolution 120,000, max injection time 50 ms, RF lens 30%; dynamic exclusion: 60 s; MS2 ion trap precursor isolation window 0.7 *m*/*z*; CID collision energy: 35%; real-time search was enabled for MS3 fragmentation using SwissProt Homo sapiens reference proteome (2021.06.20), constant modification of carbamidomethyl (Cys), TMT6plex (Lys and N-terminal) and variable modification of oxidation (Met), synchronous precursor selection: 10 precursors, precursor isolation window: 3 m/z, HCD collision energy: 55%). Peptides were searched against the SwissProt Homo sapiens reference proteome (2021.06.20), with the modification of the K-Ras protein sequence to reflect the homozygous p.G12S mutation, using MaxQuant (v.2.0.3.1, https://www.maxquant.org/). Quantification was performed with reporter ions in MS3 using unique peptides only. Fold-enrichment was calculated as the ratio of the geometric means of peptide intensities of three biological replicates between the two conditions. *P* values were calculated using unpaired, two-tailed Student’s *t*-test.

### Recombinant protein expression and purification

#### K-Ras (wild-type), K-Ras (G12S) and K-Ras (G12S) Cyslight

DNA sequences encoding human K-Ras (wild-type, amino acids 1–169), human K-Ras (G12S, amino acids 1–169), human K-Ras G12S Cyslight (G12S/C51S/C80L/C118S, amino acids 1–169) and human NF1-GRD (amino acids 1,203–1,530) were codon optimized, synthesized by Twist Biosciences and cloned into pJExpress411 vector using the Gibson assembly method^51^. The resulting constructs contain a N-terminal 6× His tag and a TEV cleavage site (ENLYFQG). The full amino acid sequences can be found in Supplementary Fig. 4. The proteins were expressed and purified following previously reported protocols^15^ Briefly, chemically competent BL21(DE3) cells were transformed with the corresponding plasmid and grown on LB agar plates containing 50 µg ml^−1^ kanamycin. A single colony was used to inoculate a culture at 37 °C, 220 r.p.m. in terrific broth containing 50 µg ml^−1^ kanamycin. When the optical density reached 0.6, the culture temperature was reduced to 20 °C, and protein expression was induced by the addition of IPTG to 1 mM. After 16 h at 20 °C, cells were pelleted by centrifugation (6,500g, 10 min) and lysed in lysis buffer (20 mM Tris 8.0, 500 mM NaCl, 5 mM imidazole) with a high-pressure homogenizer (Microfluidics). The lysate was clarified by high-speed centrifugation (19,000*g*, 15 min) and the supernatant was used in subsequent purification by immobilized metal-affinity chromatography. His-TEV tagged protein was captured with Co-TALON resin (Clontech, Takara Bio; 2 ml slurry per liter of culture) at 4 °C for 1 h with constant end-to-end mixing. ﻿The loaded beads were then washed with lysis buffer (50 ml per liter of culture) and the protein was eluted with elution buffer (20 mM Tris 8.0, 300 mM NaCl, 300 mM imidazole). To this protein solution was added His-tagged TEV protease (0.05 mg TEV per mg of Ras protein) and GDP (1 mg per mg of Ras protein), and the mixture was dialyzed against TEV cleavage buffer (20 mM Tris 8.0, 300 mM NaCl, 1 mM EDTA, 1 mM DTT) at 4 °C using a 10K molecular weight cutoff dialysis cassette until LC–MS analysis showed full cleavage (typically 16–24 h). MgCl_2_ was added to a final concentration of 5 mM, and the mixture was incubated with 1 ml of Ni-NTA (Qiagen) beads at 4 °C for 1 h to remove TEV protease, any residual His-tagged proteins and peptides. The protein solution was diluted 1:10 v/v with 20 mM Tris 8.0 and further purified using anion-exchange chromatography (HiTrapQ column, GE Healthcare Life Sciences) with a NaCl gradient of 50 to 500 mM in 20 mM Tris 8.0. Nucleotide loading was performed by mixing the ion exchange-purified protein with an excess of GDP (5 mg per liter of culture) or GppNHp (5 mg per liter of culture) and 5 mM EDTA at 23 °C for 30 min. The reaction was stopped by the addition of MgCl_2_ to 10 mM. For GppNHp, an additional calf intestine phosphatase treatment was performed as follows to ensure high homogeneity of the loaded nucleotide. The protein buffer was exchanged for phosphatase buffer (32 mM Tris 8.0, 200 mM ammonium sulfate, 0.1 mM ZnCl_2_) with a HiTrap desalting column (GE Healthcare Life Sciences). To the buffer-exchanged protein solutions, GppNHp was added to 5 mg ml^−1^, and calf intestine phosphatase (NEB) was added to 10 U ml^−1^. The reaction mixture was incubated on ice for 1 h, and MgCl_2_ was added to a final concentration of 20 mM. After nucleotide loading, the protein was concentrated using a 10K molecular weight cutoff centrifugal concentrator (Amicon-15, Millipore) to 20 mg ml^−1^ and purified by size-exclusion chromatography on a Superdex 75 10/300 GL column (GE Healthcare Life Sciences). Fractions containing pure biotinylated Ras protein were pooled, concentrated to 20 mg ml^−1^ and stored at –78 °C. In our hands, this protocol gives a typical yield of 5–15 mg per liter of culture.

#### NF1-GRD

DNA sequence encoding human NF1-GRD (amino acids 1,203–1,530) was codon optimized, synthesized by Twist Biosciences and cloned into pJExpress411 vector using the Gibson assembly method. The resulting construct contains n N-terminal 6× His tag and a TEV cleavage site (ENLYFQG). Protein was expressed and purified using the identical protocol as for the Ras proteins, except that the ion exchange and nucleotide loading steps were omitted. In our hands, this protocol gives a typical yield of 5–15 mg per liter of culture.

#### Sos^cat^

The catalytic domain of Sos (residues 466–1,049, Sos^cat^) was expressed and purified following a published protocol.^52^

### Crystallization

K-Ras(G12S) Cyslight (G12S/C51S/C80L/C118S) bound by GDP purified by size-exclusion chromatography was diluted to 100 µM in reaction buffer (20 mM HEPES 7.5, 150 mM NaCl, 1 mM MgCl_2_). For co-crystal structures, G12Si-1 or G12Si-5 was added as a 10 mM solution in DMSO to a final concentration of 200 µM. The mixture was allowed to stand at 23 °C until LC–MS analysis of the reaction mixture showed full conversion to a single covalent adduct. The reaction mixture was purified by size-exclusion chromatography (Superdex75, 20 mM HEPES 7.5, 150 mM NaCl, 1 mM MgCl_2_) and concentrated to 20 mg ml^−1^. For crystallization, 0.1 µl of the protein was mixed with 0.1 µl of well buffer. Crystals were grown at 20 °C in a 96-well plate using the hanging-drop vapor diffusion method. Maximal crystal growth was achieved after 7 d. The crystals were transferred to a cryoprotectant solution (same composition as the well solution with 20% glycerol) and flash-frozen in liquid nitrogen. The well buffers for the crystals reported in this study are as follows:

- K-Ras(G12S)•GDP: 0.1 M Tris 8.7, 0.2 M CaCl2, 25% polyethylene glycol (PEG) 4000
- K-Ras(G12S)•GDP•G12Si-1: 0.1 M sodium acetate 4.6, 30% w/v PEG MME 2000, 0.2 M ammonium sulfate
- K-Ras(G12S)•GDP•G12Si-5: 0.1 M MES 6.5, 30% PEG 4000.

### X-Ray data collection and structure determination

The dataset was collected at the Advanced Light Source beamline 8.2.2 with X-ray at a wavelength of 0.999907 Å. The dataset was indexed and integrated using iMosflm^53^, scaled with Aimless^54^, and solved by molecular replacement using Phaser^55^ in CCP4 software suite^56^. The crystal structure of GDP-bound K-Ras(G12C)-MRTX849 adduct (PDB code: 6USZ) was used as the initial model. The structure was manually refined with Coot^57^ and PHENIX^58^. Data collection and refinement statistics are listed in Supplementary Table 1.

### Chemical synthesis

Detailed protocols for chemical synthesis are provided in the Supplementary Note section of Supplementary Information.

### Reporting summary

Further information on research design is available in the Nature Research Reporting Summary linked to this article.

## Online content

Any methods, additional references, Nature Research reporting summaries, source data, extended data, supplementary information, acknowledgements, peer review information; details of author contributions and competing interests; and statements of data and code availability are available at 10.1038/s41589-022-01065-9.

## Supplementary information

Supplementary Information Supplementary Figs. 1–4, Tables 1–3 and Note (chemical synthesis).

Reporting Summary

Supplementary Data 1 Full list of proteins captured by Probe 6 with or without competition by compound **5** (Extended Data Fig. 6d).

## Data Availability

Atomic coordinates and structure factors for the reported crystal structures have been deposited with the Protein Data Bank (PDB), with the following accession numbers: K-Ras(G12S)•GDP, 7TLK; K-Ras(G12S)•GDP•G12Si-1, 7TLE; K-Ras(G12S)•GDP•G12Si-5, 7TLG. Source data of uncropped, unprocessed gel images are provided with this paper.

## Source data

Source Data Fig. 1 Unprocessed western blots for Fig. 1.
Source Data Fig. 4 Unprocessed western blots for Fig. 4.
Source Data Extended Data Fig. 6 Unprocessed gel images for Extended Data Fig. 6.
