# Supplementary material for: Chemical acylation of an acquired serine suppresses oncogenic signaling of K-Ras(G12S)
Source: Nat Chem Biol. 2022 Jul 21;18(11):1177–83. doi: 10.1038/s41589-022-01065-9 (PMC9596369; doi:10.1038/s41589-022-01065-9)
Supplement: Supplementary file 1 — Supplementary Figs. 1–4, Tables 1–3 and Note (chemical synthesis). [file 41589_2022_1065_MOESM1_ESM.pdf]

nature portfolio

https://doi.org/10.1038/s41589-022-01065-9

Supplementary information

Chemical acylation of an acquired serine suppresses oncogenic signaling of K-Ras(G12S)

In the format provided by the authors and unedited

Supporting Information for

Chemical acylation of an acquired serine suppresses oncogenic signaling of K-Ras(G12S)

Ziyang Zhang[1], Keelan Z Guiley[1], Kevan M. Shokat[1,*]

[1] Department of Cellular and Molecular Pharmacology and Howard Hughes Medical Institute, University of California, San Francisco, California

[*] Corresponding author. kevan.shokat@ucsf.edu (K.M.S.).

Table of Contents

Supplementary Figures and Tables 3

Supplementary Figure 1. Sanger sequencing of the *KRAS* Exon 2 of G12S-mutant cell lines........ 3

Supplementary Figure 2. Validation of the mutant-specific Ras(G12S) antibody. 4

Supplementary Figure 3. Source data for Supplementary Figure 2. 5

Supplementary Figure 4. Amino acid sequences of recombinant proteins used in this study. 6

Supplementary Table 1. X-ray crystallography data collection and refinement statistics 7

Supplementary Table 2. List of antibodies 8

Supplementary Table 3. List of buffer compositions 8

Supplementary Dataset 1. List of proteins enriched by click probe **6**. 10

Supplementary Note 11

Chemical Synthesis 11

Supplementary Figures and Tables

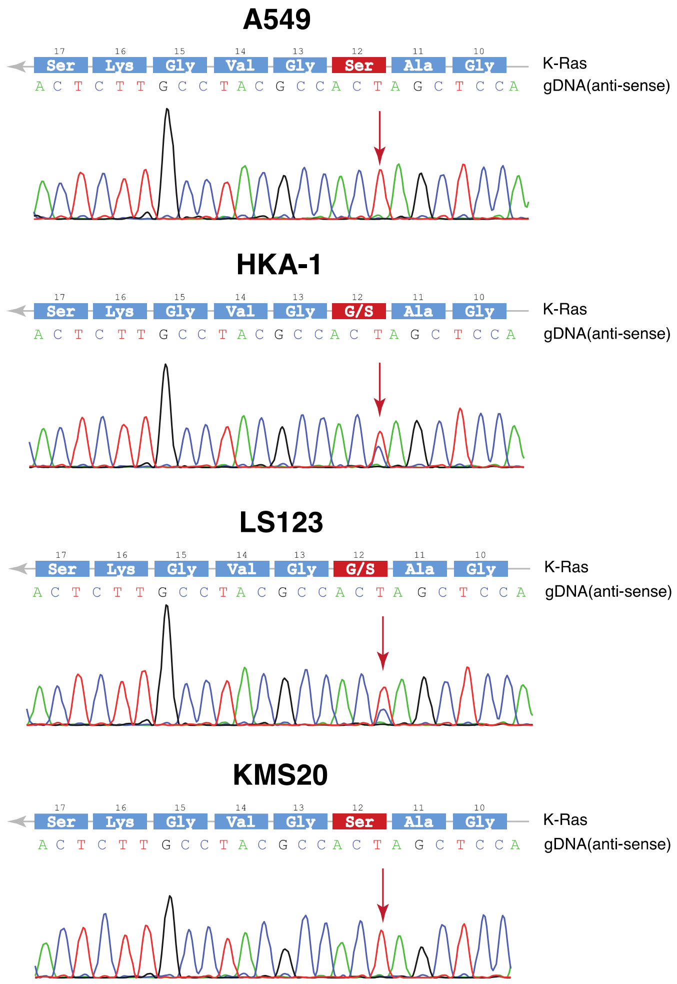

<u>Supplementary Figure 1. Sanger sequencing of the *KRAS* Exon 2 of G12S-mutant cell lines.</u> Genomic DNA sequence is presented as the antisense strand. Red arrow indicates the c. 34G>A mutation. A549 and KMS20 were determined to carry homozygous *KRAS* p. G12S mutation, and HKA-1 and LS123 were determined to carry heterozygous *KRAS* p. G12S mutation.

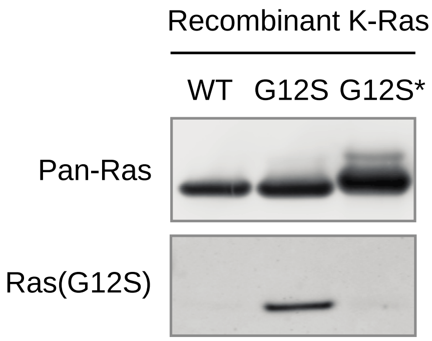

* K-Ras(G12S)•G12Si-1 adduct.

Supplementary Figure 2. Validation of the mutant-specific Ras(G12S) antibody. A pan-Ras antibody (abcam 108602) and a mutant-specific Ras(G12S) antibody (NewEastBio 26186) were used to detect recombinant K-Ras(wildtype), K-Ras(G12S) and K-Ras(G12S)•G12Si-1 adduct. Data shown is representative of two independent experiments.

Uncropped gel images for Supplementary Figure 2

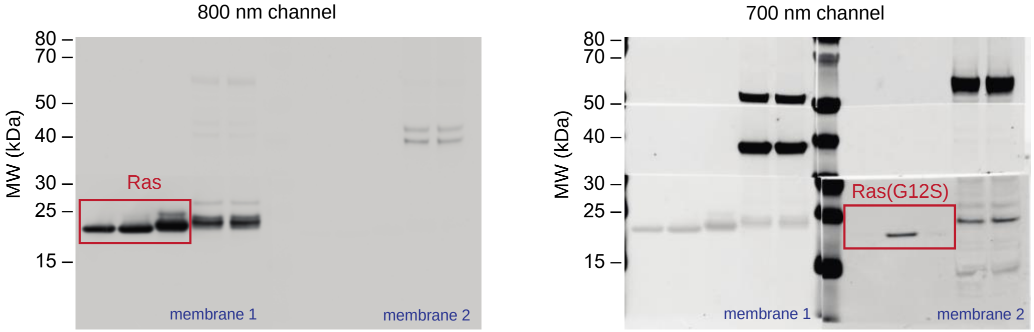

Note: Lanes 4 and 5 on each membrane contain samples from an irrelevant experiment (notebook page ZZY -BIO14-050). These bands are shown as part of a full scan image, but were not used in this manuscript.

Supplementary Figure 3. Source data for Supplementary Figure 2.

>K-Ras wildtype
MHHHHHHSSGRENLYFQG MTEYKLVVVGAGGVGKSALTI QLIQNHFVDEYDPTIEDSYRKQVVIDGETCL
LDILDTAGQEEYSAMRDQYMRTGEGFLCVFAINNTKSFE DIHHYREQIKRVKDSEDVPMVLVGNKCDLPS
RTVDTKQAQDLARSYGIPFIETSAKTRQGVDDAFYTLVR EIRKHKEK

>K-Ras wildtype CysLight
MHHHHHHSSGRENLYFQG MTEYKLVVVGASGVGKSALTI QLIQNHFVDEYDPTIEDSYRK QVVIDGETSL
LDILDTAGQEEYSAMRDQYMRTGEGFLLVFAINNTKSFE DIHHYREQIKRVKDSEDVPMVLVGNKSDLPS
RTVDTKQAQDLARSYGIPFIETSAKTRQGVDDAFYTLVR EIRKHKEK

>K-Ras G12S
MHHHHHHSSGRENLYFQG MTEYKLVVVGASGVGKSALTI QLIQNHFVDEYDPTIEDSYRKQVVIDGETCL
LDILDTAGQEEYSAMRDQYMRTGEGFLCVFAINNTKSFE DIHHYREQIKRVKDSEDVPMVLVGNKCDLPS
RTVDTKQAQDLARSYGIPFIETSAKTRQGVDDAFYTLVR EIRKHKEK

>K-Ras G12S CysLight
MHHHHHHSSGRENLYFQG MTEYKLVVVGASGVGKSALTI QLIQNHFVDEYDPTIEDSYRKQVVIDGETSL
LDILDTAGQEEYSAMRDQYMRTGEGFLLVFAINNTKSFE DIHHYREQIKRVKDSEDVPMVLVGNKSDLPS
RTVDTKQAQDLARSYGIPFIETSAKTRQGVDDAFYTLVR EIRKHKEK

>NF1-GRD
MHHHHHHSSGRENLYFQG DRFERLVELVTMMGDQGELPI AMALANVVPCSQWDELARVLVTLFDSRHLLY
QLLWNMFSKEVELADSMQ TLFRGNSLASKIMTFCFKVYG ATYLQKLLDPLLRIVITSSDWQHVSFEVDPT
RLEPSESLEENQRNLLQMTEKFFHAIISSSSEFPPQLRS VCHCLYQVVSQRFPQNSIGAVGSAMFLRFIN
PAIVSPYEAGILDKKPPPRIERGLKLMSKILQSIANHVL FTKEEHMRPFNDFVKSNFDAARRFFLDIASD
CPTSDAVNHSLSFISDGNVLALHRLLWNNQEKIGQYLSS NRDHKAVGRRPFDKMATLLAYLGPP EH

Supplementary Figure 4. Amino acid sequences of recombinant proteins used in this study. Blue test indicate affinity tag that were removed during the purification step.

Supplementary Table 1. X-ray crystallography data collection and refinement statistics

Values in parentheses are for highest resolution shell.

	K-Ras(G12S)•GDP	K-Ras(G12S)•GDP•G12Si-1	K-Ras(G12S)•GDP•G12Si-5
Data collection			
Space group	P3	$P2_12_12_1$	$P12_11$
Cell dimensions			
a, *b*, *c* (Å)	83.61, 83.61, 41.27	39.78, 50.66, 89.31	41.55, 66.53, 60.80
α, β, γ (º)	90, 90, 120	90, 90, 90	90, 101.9, 90
Resolution (Å)	72.41-1.71 (1.74-1.71)	89.31-1.99 (2.04-1.99)	66.53-1.80 (1.84-1.80)
R_{merge}	0.160 (0.280)	0.072 (0.468)	0.072 (0.232)
R_{pim}	0.083 (0.163)	0.030 (0.230)	0.043 (0.144)
I / σI	9.0 (3.9)	21.4 (3.7)	17.0 (5.8)
CC 1/2	0.987 (0.941)	0.999 (0.896)	0.997 (0.968)
Total reflections	335792 (13854)	153618 (7825)	208629 (10506)
Unique Reflections	34820 (1831)	12881 (851)	29901 (1742)
Completeness (%)	99.9 (100.0)	99.0 (95.8)	99.4 (98.6)
Redundancy	9.6 (7.6)	11.9 (9.2)	7.0 (6.0)
Refinement			
Resolution (Å)	36.2-1.71	44.65-1.99	44.35-1.80
No. reflections	34814 (3500)	25606 (2380)	29877 (2929)
R_{work} / R_{free}	15.7/18.3	18.9/23.8	18.4/23.1
No. atoms	3214	1519	3169
Protein	2724	1354	2780
Ligands	2	77	144
Refined B-factors (Å^2)			
Overall	16.67	29.86	20.06
Compound	2.26	28.65	19.28
water	27.84	34.82	30.54
R.m.s. deviations			
Bond lengths (Å)	0.010	0.013	0.011
Bond angles (°)	1.45	0.96	1.27
Ramachandran analysis			
Favored (%)	97	95	97
Disallowed (%)	1	1	0
PDB access code	7TLK	7TLE	7TLG

Supplementary Table 2. List of antibodies

Target	Supplier	Identifier	Dilution
P-AKT [S473]	Cell Signaling Technology	4060	1:1000
P-AKT [T308]	Cell Signaling Technology	4056	1:1000
AKT	Cell Signaling Technology	2920	1:1000
P-ERK [T202/Y204]	Cell Signaling Technology	9101	1:1000
Total ERK	Cell Signaling Technology	4695	1:1000
Pan-Ras	Abcam	108602	1:5000
Ras(G12S)	NewEast Bio	26186	1:1000
GAPDH	Proteintech	60004-1-Ig	1:50000

Supplementary Table 3. List of buffer compositions

Name	Composition
RIPA Buffer	25 mM Tris 7.4<br>150 mM NaCl<br>0.1% SDS<br>1% NP-40<br>0.5% sodium deoxycholate
Co-IP Lysis Buffer	50 mM HEPES 7.4<br>120 mM NaCl<br>1% NP-40<br>1 mM $MgCl_2$
Co-IP Wash Buffer	50 mM HEPES 7.4<br>120 mM NaCl<br>0.01% NP-40<br>1 mM $MgCl_2$
5x SDS Loading Buffer	250 mM Tris 6.8<br>500 mM DTT<br>10% w/v SDS<br>0.1% w/v Bromophenol Blue<br>50% Glycerol
TOWBIN Buffer	25 mM Tris<br>192 mM Glycine<br>pH 8.3
Lysis Buffer	20 mM Tris 8.0<br>500 mM NaCl<br>5 mM imidazole
Elution Buffer	20 mM Tris 8.0<br>300 mM NaCl<br>300 mM imidazole
TEV Cleavage Buffer	20 mM Tris 8.0<br>300 mM NaCl<br>1 mM EDTA

	1 mM DTT
Phosphatase Buffer	32 mM Tris 8.0<br>200 mM ammonium sulfate<br>0.1 mM $ZnCl_2$
SEC Buffer	20 mM HEPES 7.5<br>150 mM NaCl<br>1 mM $MgCl_2$
Nucleotide Exchange Buffer	20 mM HEPES 7.5<br>150 mM NaCl<br>1 mM $MgCl_2$<br>1 mM DTT
GTPase Reaction Buffer	20 mM HEPS 7.5<br>150 mM NaCl<br>1 mM DTT
5x TAMRA-N_3 Click Master Mix	5 mM CuSO4<br>1 mM TBTA<br>5% SDS<br>5 mM TCEP•HCl<br>500 µM TAMRA azide (Click Chemistry Tools AZ109)
5x Biotin-N_3 Click Master Mix	5 mM CuSO4<br>1 mM TBTA<br>5% SDS<br>5 mM TCEP•HCl<br>25 µM Biotin picolyl azide (Click Chemistry Tools 1167)

Supplementary Dataset 1. List of proteins enriched by click probe **6**.

This dataset is supplied as a separate spreadsheet file.

Supplementary Note

Chemical Synthesis

General Experiment Procedure

All reactions were performed in oven-dried glassware fitted with rubber septa under a positive pressure of argon, unless otherwise noted. Air- and moisture-sensitive liquids were transferred via syringe. Solutions were concentrated by rotary evaporation at or below 40 °C. Analytical thin-layer chromatography (TLC) was performed using glass plates pre-coated with silica gel (0.25-mm, 60-Å pore size, 230−400 mesh, Merck KGA) impregnated with a fluorescent indicator (254 nm). TLC plates were visualized by exposure to ultraviolet light (UV), then were stained by submersion in a 10% solution of phosphomolybdic acid (PMA) in ethanol or an 2% aqueous solution of potassium permanganate followed by brief heating on a hot plate. Flash column chromatography was performed with Teledyne ISCO CombiFlash EZ Prep chromatography system, employing pre-packed silica gel cartridges (Teledyne ISCO RediSep).

Solvents and Reagents

Anhydrous solvents were purchased from Acros Organics. Unless specified below, all chemical reagents were purchased from Sigma-Aldrich, AK Scientific or Chemscene. *O1*-tert-butyl *O4*-ethyl (3*R*,4*R*)-3-hydroxypiperidine-1,4-dicarboxylate was purchased from PharmaBlock Inc. Commercial solvents and reagents were used as received.

Instrumentation

Proton nuclear magnetic resonance (^{1}H NMR) spectra, carbon nuclear magnetic resonance (^{13}C NMR) spectra, and fluorine nuclear magnetic resonance (^{19}F NMR) spectra were recorded on Bruker AvanceIII HD instrument (400 MHz/100 MHz/376 MHz) at 23 °C operating with the Bruker Topspin 3.1. NMR spectra were processed using Mestrenova (version 14.1.2). Proton chemical shifts are expressed in parts per million (ppm, δ scale) and are referenced to residual protium in the NMR solvent ($CHCl_3$: δ 7.26, D_2HCOD: δ 3.31). Carbon chemical shifts are expressed in parts per million (ppm, δ scale) and are referenced to the carbon resonance of the NMR solvent ($CDCl_3$: δ 77.0, CD_3OD: δ 49.0). Fluorine chemical shifts are expressed in parts per million (ppm, δ scale) and are referenced to an external standard of trifluoroacetic acid (–76.55 ppm). Data are represented as follows: chemical shift, multiplicity (s = singlet, d = doublet, t = triplet, q = quartet, dd = doublet of doublets, dt = doublet of triplets, m = multiplet, br = broad, app = apparent), integration, and coupling constant (*J*) in Hertz (Hz). High-resolution mass spectra were obtained using a Waters Xevo G2-XS time-of-flight mass spectrometer operating with Waters MassLynx software (version 4.2).

Mini-workup

When a mini-workup (A/B) is indicated in the procedure, it was performed as follows: an aliquot (5 µL) of the reaction mixture was retrieved with a glass pipet and added to a plastic vial containing 0.2 mL organic solvent A and 0.2 mL aqueous solution B. The vial was shaken vigorously and allowed to stand until the two layers partitioned. The organic layer was then used for TLC or LC-MS analysis as specified in the procedure.

Monitoring Reaction Progress by LC-MS

When LC-MS analysis of the reaction mixture is indicated in the procedure, it was performed as follows. An aliquot (1 µL) of the reaction mixture (or the organic phase of a mini-workup mixture) was diluted with 100 µL 1:1 acetonitrile:water. 1 µL of the diluted solution was injected onto a Waters Acquity UPLC BEH C18 1.7 µm column and eluted with a linear gradient of 5–95% acetonitrile/water (+0.1% formic acid) over 3.0 min. Chromatograms were recorded with a UV detector set at 254 nm and a time-of-flight mass spectrometer (Waters Xevo G2-XS).

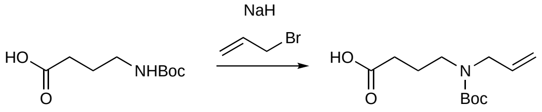

8

4-(allyl(*tert*-butoxycarbonyl)amino)butanoic acid (**8**)

A solution of 4-[(*tert*-butoxycarbonyl)amino]butanoic acid (5.00 g, 24.6 mmol, 1 equiv.) in THF (10.0 mL) was added dropwise to a suspension of sodium hydride (60% in mineral oil, 2.46 g, 2.50 equiv.) in THF (15.0 mL) at 23 ºC. After gas evolution had subsided, allyl bromide (3.19 mL, 36.9 mmol, 1.50 equiv.) was added dropwise via syringe. The resulting mixture was stirred at 23 ºC for 24 h. 10% Citric acid was added dropwise until the pH of the solution reached 5. Care must be taken at the beginning of the addition as the excess sodium hydride may react violently with water if the solution is added too fast. The reaction mixture was extracted with ether (3 x 20 mL). The combined ether layers were washed with water (2 x 30 mL) and saturated aqueous sodium chloride solution (30 mL). The washed solution was dried over magnesium sulfate, and the dried solution was concentrated. The residue was purified by column chromatography (20–50% ethyl acetate–hexanes) to afford the product as a colorless oil (4.70 g, 78.5%).

^{1}H NMR (400 MHz, $CDCl_3$) δ 5.79 (ddt, *J* = 16.3, 11.0, 5.7 Hz, 1H), 5.19 – 5.10 (m, 2H), 3.82 (br s, 2H), 3.30 (br s, 2H), 2.39 (t, *J* = 7.1 Hz, 2H), 1.87 (p, *J* = 7.0 Hz, 2H), 1.48 (s, 9H).
^{13}C NMR (100 MHz, $CDCl_3$) δ 178.53, 155.70 (br), 133.93, 116.55 (br), 79.93, 49.52 (br), 45.63, 31.22 (br), 28.34, 23.31.
HRMS ($C_{12}H_{21}NO_4$ + Na)$^+$ Calc'd: 266.1368, Found: 266.1383.

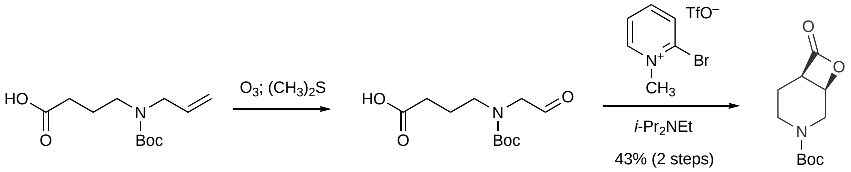

8 **9** (±)-**10**

4-((*tert*-butoxycarbonyl)(2-oxoethyl)amino)butanoic acid (**9**)

A solution of **S1** (4.70 g, 19.3 mmol, 1 equiv.) in dichloromethane (96.5 mL) was cooled to –78 ºC (dry ice/acetone bath). A stream of ozone was bubbled through the solution until a persistent pale blue color was observed. Dimethyl sulfide (7.09 mL, 96.5 mmol, 5.00 equiv.) was added to the reaction mixture, and the solution was allowed to warm to 23 ºC. The resulting mixture was stirred for 24 h at 23 ºC. The reaction mixture was directly washed with water (100 mL), and the washed organic layer was dried over sodium sulfate. The dried solution was filtered and

concentrated under reduced pressure to afford the product as a colorless oil. The product was used without further purification.

Rac-tert-butyl (1*R*,6*R*)-7-oxo-8-oxa-3-azabicyclo[4.2.0]octane-3-carboxylate (±-**10**)

A solution of **9** (800 mg, 3.26 mmol, 1 equiv.) in acetonitrile (32.62 mL) was added dropwise over 1 h to a stirred solution of 2-bromo-1-methylpyridinium trifluoromethanesulfonate (3.15 g, 9.78 mmol, 3.00 equiv.), and triethylamine (1.82 mL, 13.05 mmol, 4.00 equiv.) in acetonitrile (32.6 mL) at 23 ºC. The resulting mixture was further stirred at 23 ºC for 22 h. The reaction mixture was directly concentrated, and the residue was purified by column chromatography (30–100% ethyl acetate–hexanes) to afford the product as a colorless oil, which solidified upon standing (322 mg, 43.4%).

^{1}H NMR (1:1 mixture of rotamers, some peaks are resolved for the two rotamers, 400 MHz, $CDCl_3$) δ 4.82 (br s, 0.5 H), 4.77 (br s, 0.5H), 4.41 (br d, *J* = 15.6 Hz, 0.5H), 4.28 (br d, *J* = 15.6 Hz, 0.5H), 3.90 – 3.82 (m, 1H), 3.61 (s, 1H), 3.52 – 3.28 (m, 2H), 2.25 – 2.06 (m, 1H), 2.06 – 1.86 (m, 1H), 1.48 (s, 9H).

^{13}C NMR (1:1 mixture of rotamers, some peaks are resolved for the two rotamers, 100 MHz, $CDCl_3$) δ 169.66 (br), 155.27 (br), 80.41, 69.32 (br), 68.97 (br), 47.54 (br), 42.32 (br), 40.87 (br), 40.06 (br), 39.15(br), 28.38, 19.83.

HRMS $(C_{11}H_{17}NO_4 + H – tBu)^+$ Calc'd: 172.0610, Found: 172.0621

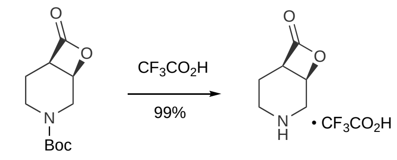

(±)-**10** (±)-**11**

Rac-(1*R*,6*R*)-8-oxa-3-azabicyclo[4.2.0]octan-7-one trifluoroacetate (±-**11**)

(±)-**10** (200 mg, 0.880 mmol, 1 equiv.) was dissolved in 1:1 trifluoroacetic acid:dichloromethane (1.0 mL). The resulting yellow solution was allowed to stand at 0 ºC for 1 h, then was concentrated *in vacuo*. The resulting yellow oil was triturated with anhydrous ether (10 mL) and the resulting solids were collected by centrifugation. Drying under vacuum afforded the product as a yellow powder (210 mg, 99.0%).

^{1}H NMR (400 MHz, MeOD) δ 4.99 (ddd, *J* = 5.9, 3.4, 2.0 Hz, 1H), 4.11 (td, *J* = 6.8, 3.8 Hz, 1H), 3.73 (dd, *J* = 15.1, 2.1 Hz, 1H), 3.58 (dd, *J* = 15.1, 3.4 Hz, 1H), 3.38 – 3.20 (m, 2H), 2.35 – 2.13 (m, 2H).

^{13}C NMR (100 MHz, MeOD) δ 169.33, 65.77, 45.81, 41.15, 38.29, 16.72. Signals from the trifluoroacetate anion were too weak to be observed.

^{19}F NMR (376 MHz, MeOD) δ -76.92.

HRMS $(C_6H_9NO_2 + H)^+$ Calc'd: 128.0712, Found: 128.0707

Enantiomerically pure (1*R*,6*R*)-**11** was prepared from the commercial starting material *O1-tert*-butyl *O4*-ethyl (3*R*,4*R*)-3-hydroxypiperidine-1,4-dicarboxylate.

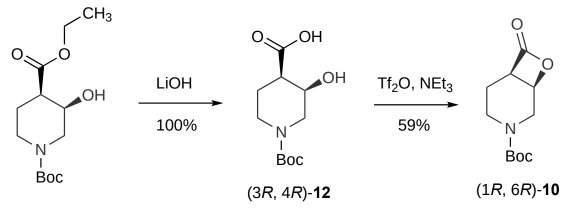

(3*R*,4*R*)-1-(*tert*-butoxycarbonyl)-3-hydroxypiperidine-4-carboxylic acid (3*R*,4*R*-**12**)
An aqueous solution of lithium hydroxide hydrate (5.49 mL, 5.49 mmol, 1.50 equiv.) was added to a stirred solution of *O1*-*tert*-butyl *O4*-ethyl (3*R*,4*R*)-3-hydroxypiperidine-1,4-dicarboxylate (1.00 g, 3.66 mmol) in THF (7.7 mL). After 30 min, 10% citric acid (10 mL) was added, and the reaction mixture was extracted with ether (3 x 10 mL). The combined organic layers were washed with saturated sodium chloride solution, the washed solution was dried over magnesium sulfate, and the dried solution was concentrated to afford the product as a white crystalline solid (0.90 g, 100%).

^{1}H NMR (400 MHz, $CDCl_3$) δ 4.25 (s, 1H), 4.20 – 4.02 (m, 2H), 3.04 (d, *J* = 14.0 Hz, 1H), 2.95 – 2.83 (m, 1H), 2.64 (ddd, *J* = 11.7, 4.3, 2.6 Hz, 1H), 2.18 – 2.03 (m, 1H), 1.80 (dd, *J* = 13.9, 3.9 Hz, 1H), 1.49 (s, 9H).
HRMS ($C_{11}H_{19}NO_5$ – H)$^{-}$ Calc'd: 244.1185, Found: 244.1185.

tert-butyl (1*R*,6*R*)-7-oxo-8-oxa-3-azabicyclo[4.2.0]octane-3-carboxylate ((1*R*,6*R*)-**10**)

Triflic anhydride (71.9 µL, 0.428 mmol, 1.05 equiv.) was added to a stirred solution of (3*R*,4*R*)-**12** (100 mg, 0.408 mmol, 1 equiv.) and triethylamine (170 µL, 1.22 mmol, 3.00 equiv.) in DCM (4.1 mL) at 0 ºC. The resulting mixture was stirred at 0 ºC for 1 h, then was stored without stirring at 4 ºC for 16 h. The reaction mixture was partitioned between saturated aqueous sodium bicarbonate solution (5 mL) and dichloromethane (5 mL). The layers were separated, and the aqueous layer was extracted with dichloromethane (2 x 5 mL). The combined organic layers were dried over sodium sulfate. The dried solution was filtered, and the filtrate was concentrated. The residue was purified by column chromatography (20–100% ethyl acetate–hexanes, 4-g RediSep(R) Rf column, Teledyne ISCO, Lincoln, NE) to afford the product ((1*R*,6*R*)-**10**) as a white crystalline solid (55 mg, 59%).

Spectral data was identical to (±)-**10.**

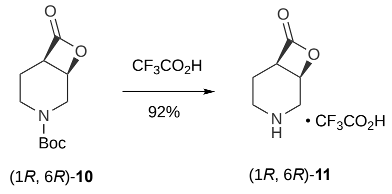

(1*R*,6*R*)-8-oxa-3-azabicyclo[4.2.0]octan-7-one trifluoroacetate ((1*R*,6*R*)-**11**)

(1*R*,6*R*)-**10** (100 mg, 0.440 mmol, 1 equiv.) was dissolved in 1:1 trifluoroacetic acid:dichloromethane (1.0 mL). The resulting yellow solution was allowed to stand at 0 ºC for 1

h, then was concentrated *in vacuo*. The resulting yellow oil was triturated with anhydrous ether (10 mL) and the resulting solids were collected by centrifugation. Drying under vacuum afforded the product ((1*R*,6*R*)-**11**) as a yellow powder (97 mg, 92%).

Spectral data was identical to (±)-**11.**
Enantiomeric excess: >99%.

Racemate standard:

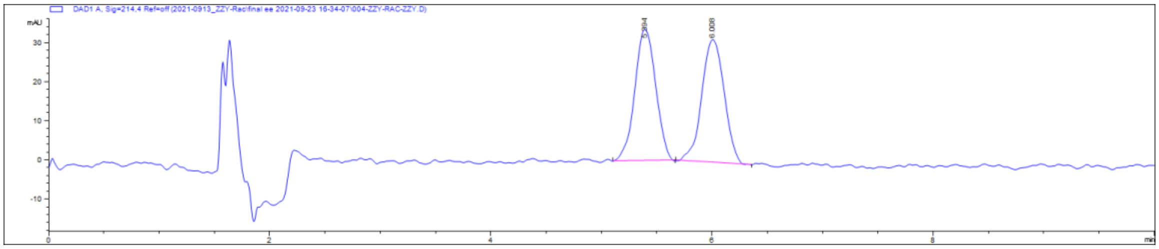

ZZY-14-015 (sample):

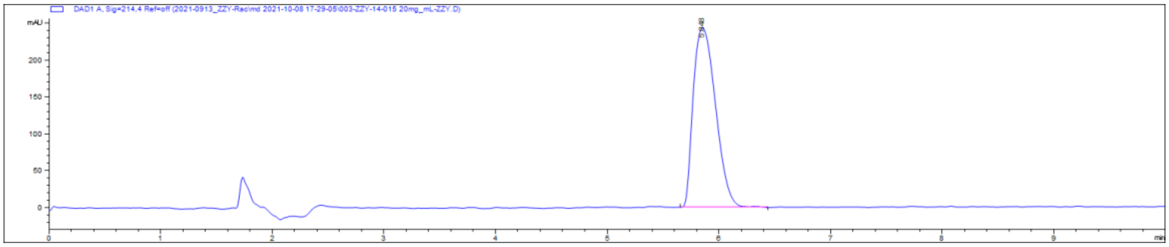

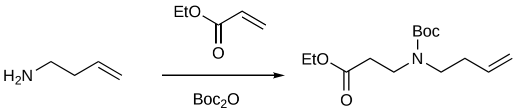

13

<u>Ethyl 3-(but-3-en-1-yl(*tert*-butoxycarbonyl)amino)propanoate (**13**)</u>

1-amino-3-butene hydrochloride (538 mg, 5 mmol, 1 equiv.) was added dropwise to a solution of ethyl acrylate (500 mg, 5.00 mmol, 1.00 equiv.) and triethylamine (697 µL, 5.00 mmol, 1.00 equiv) in THF (5.0 mL) at 23 ºC. The resulting mixture was stirred at 23 ºC. In 16 h, TLC analysis (100% ethyl acetate) showed full consumption of the amine starting material. The reaction mixture was concentrated in vacuo. THF (10 mL) was added to the residue, followed by triethylamine (697 µL, 5.00 mmol, 1.00 equiv.) and Di-*tert*-butyl dicarbonate (1.09 g, 5.00 mmol, 1.00 equiv.). Gas evolution immediately ensued and subsided over ~20 min. The reaction mixture was concentrated, and the residue was purified by column chromatography (20–50% ethyl acetate–hexanes) to afford the intermediate Michael addition product as a colorless oil (650 mg, 47.9%).

^{1}H NMR (400 MHz, $CDCl_3$) δ 5.78 (td, *J* = 17.0, 6.9 Hz, 1H), 5.17 – 5.00 (m, 2H), 4.16 (q, *J* = 7.1 Hz, 2H), 3.48 (s, 2H), 3.28 (s, 2H), 2.58 (s, 2H), 2.35 – 2.25 (m, 2H), 1.48 (s, 9H), 1.28 (t, *J* = 7.1 Hz, 3H).

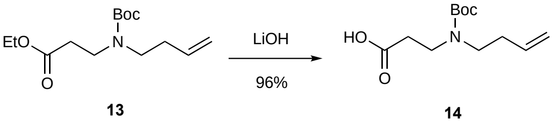

3-(but-3-en-1-yl(*tert*-butoxycarbonyl)amino)propanoic acid (**14**)

Lithium hydroxide hydrate (64.8 mg, 1.54 mmol) was added to a solution of **13** (209 mg, 0.772 mmol, 1 equiv.) in 1:1 THF (1.5 mL):Water (1.5 mL) at 23 ºC. The resulting biphasic mixture was stirred at that temperature for 4 h. TLC analysis (50% ethyl acetate–hexanes) at this point showed full consumption of the ester starting material. 10% Citric acid (10 mL) was added to the reaction mixture. The reaction mixture was extracted with ether (3 x 10 mL). The combined organic layers were dried over magnesium sulfate. The dried solution was filtered, and the filtrate was concentrated to afford the product as a colorless oil (180 mg, 95.8%).

^{1}H NMR (400 MHz, $CDCl_3$) δ 5.78 (ddt, *J* = 17.1, 10.2, 6.9 Hz, 1H), 5.13 – 5.02 (m, 2H), 3.50 (t, *J* = 6.9 Hz, 2H), 3.31 (t, *J* = 7.3 Hz, 2H), 2.67 (s, 2H), 2.29 (p, *J* = 7.3 Hz, 2H), 1.49 (s, 9H). HRMS $(C_{12}H_{21}NO_4 + Na)^+$ Calc'd: 266.1368, Found: 266.1383

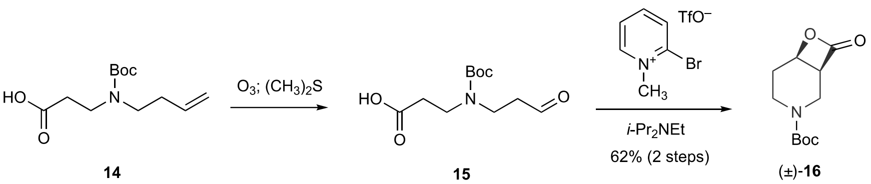

3-((*tert*-butoxycarbonyl)(3-oxopropyl)amino)propanoic acid (**15**)

Ozone was bubbled through a solution of **14** (246 mg, 1.01 mmol, 1 equiv.) in dichloromethane (4.52 mL) at –78 ºC until a purple color persisted. The gas source was switched to argon, and bubbling was continued for 10 min to remove excess ozone. Dimethyl sulfide (0.74 mL, 10.1 mmol, 10.0 equiv.) was added, and the reaction mixture was allowed to warm to 23 ºC and kept stirred at that temperature for 15 h. The reaction mixture was partitioned between water (5 mL) and dichloromethane (5 mL). The layers were separated, and the aqueous layer was extracted with dichloromethane (2 x 5 mL). The combined organic layers were dried over sodium sulfate. The dried solution was filtered, and the filtrate was concentrated to afford the product as a yellow oil. The product was used in the next reaction without further purification.

Rac-*tert*-butyl (1*S*,6*R*)-8-oxo-7-oxa-3-azabicyclo[4.2.0]octane-3-carboxylate (±-**16**)

A flame-dried 100-ml flask was charged with 2-bromo-1-methylpyridinium trifluoromethanesulfonate (969 mg, 3.01 mmol, 3.00 equiv), acetonitrile (17.1 mL), triethylamine (0.56 mL, 4.012 mmol, 4.00 equiv.) and a magnetic stir bar. A solution of **15** (246 mg, 1.00 mmol, 1 equiv.) in acetonitrile (6 mL) was added with a syringe pump at rate of 2 mL/min. After addition, the reaction solution was kept stirred at 23 ºC for 16 h. The reaction mixture was concentrated in vacuo. The residue was partitioned between 0.5 M pH 7 phosphate buffer (10 mL) and dichloromethane (10 mL). The layers were separated, and the aqueous layer was extracted with dichloromethane (2 x 10 mL). The combined organic layers were dried over sodium sulfate, and the dried solution was concentrated. The residue was purified by column chromatography (10–100% ethyl acetate–hexanes) to afford the product as a colorless oil, which solidified upon standing (141 mg, 61.9%).

^{1}H NMR (1:1 mixture of rotamers, some peaks are resolved for the two rotamers, 400 MHz, $CDCl_3$) δ 4.89 (dt, *J* = 6.0, 2.8 Hz, 1H), 4.24 – 4.08 (m, 1H), 3.90 (s, 0.5H), 3.84 (s, 0.5H), 3.63 – 3.44 (m, 2H), 3.63 – 3.44 (m, 2H), 3.38 – 3.25 (m, 1H), 2.42 – 2.25 (m, 1H), 2.18 – 2.01 (m, 1H), 1.45 (s, 9H).
^{13}C NMR (100 MHz, $CDCl_3$) δ 169.80, 154.66 (br), 80.38 (br), 68.40, 68.27, 50.16, 49.84, 37.99, 36.95, 36.46, 35.68, 28.34, 25.31.
HRMS ($C_{11}H_{17}NO_4$ + Na)$^+$ Calc'd: 250.1055, Found: 250.1060.

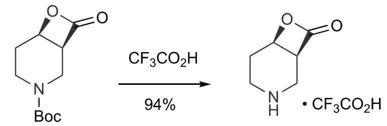

(±)-**16** (±)-**17**

<u>*Rac*-(1*S*,6*R*)-7-oxa-3-azabicyclo[4.2.0]octan-8-one (±-**17**)</u>

Rac-*tert*-butyl (1*S*,6*R*)-8-oxo-7-oxa-3-azabicyclo[4.2.0]octane-3-carboxylate (±)-**16** (200 mg, 0.880 mmol, 1 equiv.) was dissolved in 1:1 trifluoroacetic acid:dichloromethane (1.0 mL). The resulting yellow solution was allowed to stand at 0 ºC for 1 h, then was concentrated in vacuo. The resulting yellow oil was triturated with anhydrous ether (10 mL) and the resulting solids were collected by centrifugation. Drying under vacuum afforded the product as a yellow powder (200 mg, 94.2%).

^{1}H NMR (400 MHz, MeOD) δ 5.02 (ddd, *J* = 7.1, 4.3, 3.0 Hz, 1H), 4.20 (ddd, *J* = 7.5, 6.7, 2.8 Hz, 1H), 3.59 (dd, *J* = 14.2, 2.8 Hz, 1H), 3.47 (dd, *J* = 14.2, 7.5 Hz, 1H), 3.35 – 3.23 (m, 2H), 2.48 – 2.32 (m, 2H).
^{13}C NMR (100 MHz, MeOD) δ 168.92, 66.53, 44.69, 36.39, 35.77, 23.48. Signals from the trifluoroacetate anion were too weak to be observed.
^{19}F NMR (376 MHz, MeOD) δ -76.92.
HRMS ($C_6H_9NO_2$ + H)$^+$ Calc'd: 128.0712, Found: 128.0707.

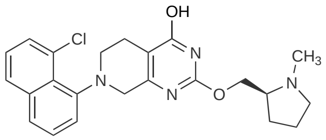

18

7-(8-chloro-1-naphthyl)-2-[[(2*S*)-1-methylpyrrolidin-2-yl]methoxy]-6,8-dihydro-5H-pyrido[3,4-d]pyrimidin-4-ol (**18**) was prepared according to the protocol described in US Patent App. 2020/0331911.

^{1}H NMR (400 MHz, MeOD) δ 8.37 (s, 2H), 7.79 (d, *J* = 8.1 Hz, 1H), 7.64 (d, *J* = 8.1 Hz, 1H), 7.50 (d, *J* = 7.4 Hz, 1H), 7.44 (t, *J* = 7.8 Hz, 1H), 7.40 – 7.31 (m, 1H), 7.27 (dd, *J* = 7.5, 2.3 Hz, 1H), 4.73 (td, *J* = 13.1, 3.1 Hz, 1H), 4.61 – 4.50 (m, 1H), 4.04 (dd, *J* = 17.5, 2.8 Hz, 1H), 3.86 – 3.67 (m, 2H), 3.62 (dd, *J* = 17.4, 2.5 Hz, 1H), 3.48 (dd, *J* = 12.6, 5.6 Hz, 1H), 3.19 (dt, *J* = 11.2, 8.0 Hz, 1H), 3.09 (dd, *J* = 10.6, 4.0 Hz, 1H), 3.04 (s, 3H), 2.89 – 2.76 (m, 1H), 2.54 (d, *J* = 16.4 Hz, 1H), 2.40 – 2.26 (m, 1H), 2.22 – 2.03 (m, 2H), 2.03 – 1.89 (m, 1H).

^{13}C NMR (100 MHz, MeOD) δ 166.56, 165.02, 158.63, 154.85, 154.82, 148.37, 148.33, 137.51, 129.53, 129.30, 128.17, 126.32, 125.71, 125.70, 125.34, 124.88, 124.85, 118.67, 118.63, 113.05, 113.03, 66.84, 65.02, 57.03, 56.91, 56.85, 49.61, 49.51, 40.01, 26.18, 26.16, 21.98, 21.95, 21.84, 21.78.
HRMS $(C_{23}H_{25}ClN_4O_2 + H)^+$ Calc'd: 425.1744, Found: 425.1732.

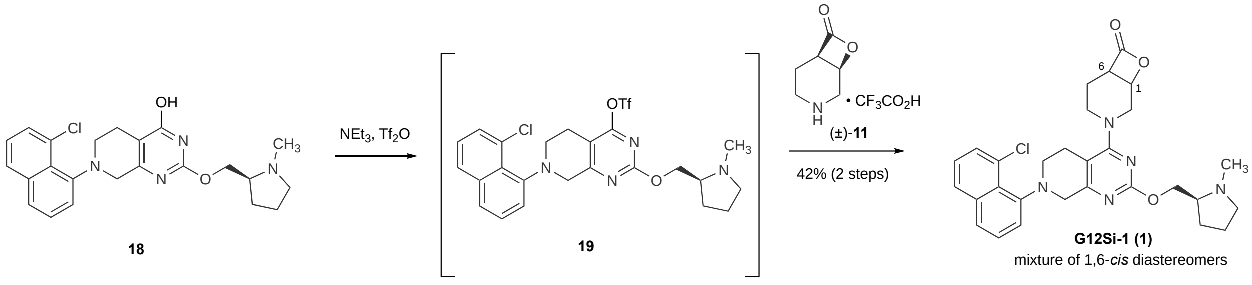

<u>G12Si-1 (**1**) (mixture of two 1,6-*cis* diastereomers).</u>
Triethylamine (29.5 µL, 0.212 mmol, 3.00 equiv.) and triflic anhydride (14.2 µL, 0.0850 mmol, 1.20 equiv.) were added sequentially to a stirred solution of **18** (30.0 mg, 0.0710 mmol, 1 equiv.) in dichloromethane (0.18 mL) at 0 ºC. In 15 min, TLC analysis (10% methanol–dichloromethane) showed full conversion of the starting material to a slightly less polar spot. The reaction mixture was partitioned between saturated aqueous sodium bicarbonate solution (5 mL) and dichloromethane (5 mL). The layers were separated, and the aqueous layer was extracted with dichloromethane (2 x 5 mL). The combined organic layers were dried over sodium sulfate. The dried solution was filtered, and the filtrate was concentrated to afford the triflate **19** as a crude product. DMF (0.350 mL) was added to the residue. (±)-**11** (34.0 mg, 0.141 mmol, 2.00 equiv.) was added to the resulting solution as a solid. Triethylamine (29.5 µL, 0.212 mmol, 3.00 equiv.) was added via syringe. The resulting brown mixture was stirred at 23 ºC for 1 h. The residue was diluted with 50% acetonitrile–water to a volume of 2.0 mL, and the solution was filtered through a 0.45 µM PTFE syringe filter. The filtrate was loaded onto a Redi-Sep C18 30g Gold column equilibrated with 10% acetonitrile–water + 0.1% formic acid, and the product was purified by column chromatography (10–100% acetonitrile–water + 0.1% formic acid). The product-containing fractions were lyophilized to afford the product as a white solid (15.7 mg, 41.6%).
^{1}H NMR (400 MHz, $CDCl_3$) δ 7.80 – 7.72 (m, 1H), 7.67 – 7.56 (m, 1H), 7.56 – 7.40 (m, 2H), 7.39 – 7.32 (m, 1H), 7.27 – 7.16 (m, 1H), 5.04 – 4.81 (m, 1H), 4.49 – 4.30 (m, 3H), 4.22 – 4.12 (m, 1H), 4.06 – 3.68 (m, 3H), 3.66 – 3.43 (m, 2H), 3.39 – 2.90 (m, 3H), 2.87 – 2.58 (m, 2H), 2.54 – 2.46 (m, 3H), 2.41 – 2.23 (m, 2H), 2.16 – 2.00 (m, 1H), 1.90 – 1.73 (m, 3H).
Note: Product was a mixture of diastereomers. NMR peaks were reported as seen.
HRMS $(C_{29}H_{32}ClN_5O_3 + H)^+$ Calc'd: 534.2272, Found: 534.2305.

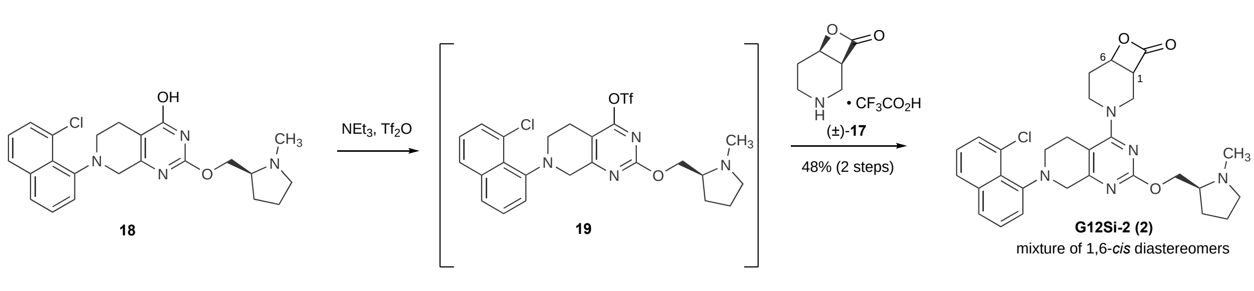

G12Si-2 (**2**) (mixture of two 1,6-*cis* diastereomers).

Triethylamine (29.5 µL, 0.212 mmol, 3.00 equiv.) and triflic anhydride (14.2 µL, 0.0850 mmol, 1.20 equiv.) were added sequentially to a stirred solution of **18** (30.0 mg, 0.0710 mmol, 1 equiv.) in DCM (0.18 mL) at 0 ºC. In 15 min, TLC analysis (10% methanol–dichloromethane) showed full conversion of the starting material to a slightly less polar spot. The reaction mixture was partitioned between saturated aqueous sodium bicarbonate solution (5 mL) and dichloromethane (5 mL). The layers were separated, and the aqueous layer was extracted with dichloromethane (2 x 5 mL). The combined organic layers were dried over sodium sulfate. The dried solution was filtered, and the filtrate was concentrated to afford the triflate **19** as a crude product. DMF (0.350 mL) was added to the residue. (±)-**17** (34.0 mg, 0.141 mmol, 2.00 equiv.) was added to the resulting solution as a solid. Triethylamine (29.5 µL, 0.212 mmol, 3.00 equiv.) was added via syringe. The resulting brown mixture was stirred at 23 ºC for 1 h. The residue was diluted with 50% acetonitrile–water to a volume of 2.0 mL, and the solution was filtered through a 0.45 µM PTFE syringe filter. The filtrate was loaded onto a Redi-Sep C18 30g Gold column equilibrated with 10% acetonitrile–water + 0.1 formic acid, and the product was purified by column chromatography (10–100% acetonitrile–water + 0.1% formic acid). The product-containing fractions were lyophilized to afford the product as a white solid (18.0 mg, 47.7%).

^{1}H NMR (400 MHz, $CDCl_3$) δ 7.79 – 7.72 (m, 1H), 7.66 – 7.58 (m, 1H), 7.58 – 7.40 (m, 2H), 7.37 – 7.31 (m, 1H), 7.27 – 7.12 (m, 1H), 5.03 – 4.92 (m, 1H), 4.52 – 4.34 (m, 3H), 4.28 – 4.14 (m, 1H), 4.13 – 3.95 (m, 2H), 3.94 – 3.69 (m, 1H), 3.69 – 3.45 (m, 2H), 3.43 – 2.88 (m, 4H), 2.85 – 2.59 (m, 2H), 2.59 – 2.50 (m, 3H), 2.50 – 2.16 (m, 2H), 2.16 – 2.01 (m, 2H), 2.01 – 1.72 (m, 3H).
Note: Product was a mixture of diastereoisomers. NMR peaks were reported as seen.
HRMS ($C_{29}H_{32}ClN_5O_3$ + H)$^+$ Calc'd: 534.2272, Found: 534.2305.

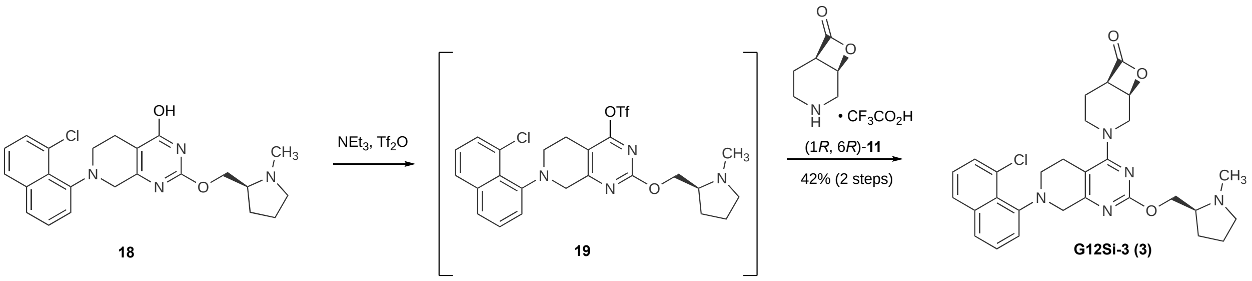

G12Si-3 (**3**).

Triethylamine (29.5 µL, 0.212 mmol, 3.00 equiv.) and triflic anhydride (14.2 µL, 0.0850 mmol, 1.20 equiv.) were added sequentially to a stirred solution of **18** (30.0 mg, 0.0710 mmol, 1 equiv.) in DCM (0.18 mL) at 0 ºC. In 15 min, TLC analysis (10% methanol–dichloromethane) showed full conversion of the starting material to a slightly less polar spot. The reaction mixture was partitioned between saturated aqueous sodium bicarbonate solution (5 mL) and dichloromethane (5 mL). The layers were separated, and the aqueous layer was extracted with dichloromethane (2 x 5 mL). The combined organic layers were dried over sodium sulfate. The dried solution was filtered, and the filtrate was concentrated to afford the triflate **19** as a crude product. DMF (0.350 mL) was added to the residue. (1*R*,6*R*)-**11** (34.0 mg, 0.141 mmol, 2.00 equiv.) was added to the resulting solution as a solid. Triethylamine (29.5 µL, 0.212 mmol, 3.00 equiv.) was added via syringe. The resulting brown mixture was stirred at 23 ºC for 1 h. The

residue was diluted with 50% acetonitrile–water to a volume of 2.0 mL, and the solution was filtered through a 0.45 µM PTFE syringe filter. The filtrate was loaded onto a Redi-Sep C18 30g Gold column equilibrated with 10% acetonitrile–water + 0.1% formic acid, and the product was purified by column chromatography (10–100% acetonitrile–water + 0.1% formic acid). The product-containing fractions were lyophilized to afford the product as a white solid (16.0 mg, 42.4%).

^{1}H NMR (400 MHz, $CDCl_3$) δ 7.80 – 7.70 (m, 1H), 7.67 – 7.58 (m, 1H), 7.57 – 7.41 (m, 2H), 7.35 (app t, *J* = 7.8 Hz, 1H), 7.27 – 7.14 (m, 1H), 4.97 – 4.81 (m, 1H), 4.49 – 4.34 (m, 3H), 4.22 – 4.09 (m, 1H), 4.01 – 3.89 (m, 2H), 3.89 – 3.67 (m, 2H), 3.64 – 3.39 (m, 2H), 3.38 – 3.18 (m, 1H), 3.18 – 2.87 (m, 2H), 2.81 – 2.58 (m, 2H), 2.50 (app d, 3H), 2.41 – 2.22 (m, 2H), 2.16 – 2.01 (m, 2H), 1.92 – 1.70 (m, 3H).
Note: Product appears as a mixture of conformational isomers. NMR peaks were reported as seen.
HRMS ($C_{29}H_{32}ClN_5O_3$ + H)$^+$ Calc'd: 534.2272, Found: 534.2257.

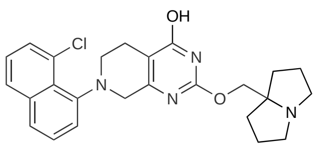

20

7-(8-chloronaphthalen-1-yl)-2-((tetrahydro-1H-pyrrolizin-7a(5H)-yl)methoxy)-5,6,7,8-tetrahydropyrido[3,4-d]pyrimidin-4-ol (**20**) was prepared according to the protocol described for compound **18**, except where (2*S*)-1-methylpyrrolidine was substituted for 1,2,3,5,6,7-hexahydropyrrolizin-8-ylmethanol.

^{1}H NMR (formate salt, 400 MHz, MeOD) δ 8.30 (s, 2H), 7.83 (dd, *J* = 8.2, 1.3 Hz, 1H), 7.68 (dd, *J* = 8.2, 1.2 Hz, 1H), 7.56 – 7.44 (m, 2H), 7.42 – 7.31 (m, 2H), 4.63 – 4.43 (m, 2H), 4.07 (d, *J* = 17.4 Hz, 1H), 3.75 – 3.62 (m, 3H), 3.55 (ddt, *J* = 11.9, 5.9, 2.1 Hz, 1H), 3.28 (dt, *J* = 12.1, 6.5 Hz, 2H), 3.17 (ddd, *J* = 11.9, 10.3, 4.1 Hz, 1H), 2.93 – 2.79 (m, 1H), 2.60 (ddt, *J* = 16.6, 4.1, 2.0 Hz, 1H), 2.32 – 2.02 (m, 8H).
^{13}C NMR (formate salt,100 MHz, MeOD) δ 165.60, 158.74, 155.07, 148.38, 137.55, 129.53, 129.28, 128.16, 126.31, 125.72, 125.33, 124.86, 118.63, 113.18, 79.65, 68.77, 57.12, 55.30, 49.48, 33.98, 33.96, 23.74, 21.85.
HRMS ($C_{25}H_{27}ClN_4O_2$ + H)$^+$ Calc'd: 451.1901, Found: 451.1911.

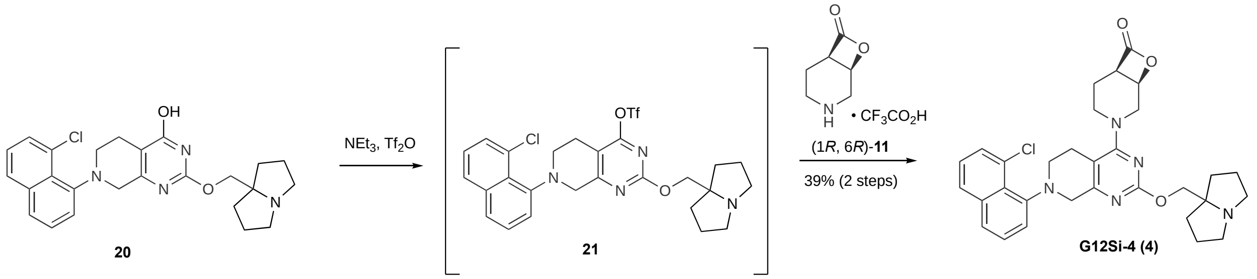

<u>G12Si-4 (**4**).</u>

Triethylamine (29.5 µL, 0.212 mmol, 3.00 equiv.) and triflic anhydride (14.2 µL, 0.0850 mmol, 1.20 equiv.) were added sequentially to a stirred solution of **20** (30.0 mg, 0.0710 mmol, 1 equiv.) in dichloromethane (0.18 mL) at 0 ºC. In 15 min, TLC analysis (10% methanol–dichloromethane) showed full conversion of the starting material to a slightly less polar spot. The reaction mixture was partitioned between saturated aqueous sodium bicarbonate solution (5 mL) and dichloromethane (5 mL). The layers were separated, and the aqueous layer was extracted with dichloromethane (2 x 5 mL). The combined organic layers were dried over sodium sulfate. The dried solution was filtered, and the filtrate was concentrated to afford the triflate **21** as a crude product. DMF (0.350 mL) was added to the residue. (1*R*,6*R*)-**11** (34.0 mg, 0.141 mmol, 2.00 equiv.) was added to the resulting solution as a solid. Triethylamine (29.5 µL, 0.212 mmol, 3.00 equiv.) was added via syringe. The resulting brown mixture was stirred at 23 ºC for 1 h. The residue was diluted with 50% acetonitrile–water to a volume of 2.0 mL, and the solution was filtered through a 0.45 µM PTFE syringe filter. The filtrate was loaded onto a Redi-Sep C18 30g Gold column equilibrated with 10% acetonitrile–water + 0.1% formic acid, and the product was purified by column chromatography (10–100% acetonitrile–water + 0.1% formic acid). The product-containing fractions were lyophilized to afford the product as a white solid (14.7 mg, 39.0%).
^{1}H NMR (formate salt, 400 MHz, MeOD) δ 7.83 (ddd, *J* = 8.2, 3.2, 1.2 Hz, 1H), 7.68 (td, *J* = 8.3, 1.2 Hz, 1H), 7.57 – 7.42 (m, 2H), 7.42 – 7.24 (m, 2H), 4.98 – 4.91 (m, 1H), 4.50 – 4.24 (m, 4H), 4.06 – 3.96 (m, 1H), 3.93 – 3.75 (m, 1H), 3.75 – 3.46 (m, 3H), 3.47 – 3.37 (m, 2H), 3.26 – 2.90 (m, 4H), 2.86 – 2.56 (m, 2H), 2.41 – 1.84 (m, 10H).
^{13}C NMR (formate salt, 100 MHz, MeOD) δ 171.40, 168.86, 166.52, 164.39, 161.57, 148.47, 137.49, 129.51, 129.37, 128.17, 126.42, 125.71, 125.37, 124.96, 118.57, 107.77, 76.95, 70.06, 69.83, 59.34, 55.17, 49.83, 46.17, 44.00, 43.81, 34.76, 26.87, 25.74, 24.08, 19.51, 18.99.
Note: Product appears as a mixture of conformational isomers. NMR peaks were reported as seen.
HRMS $(C_{31}H_{34}ClN_5O_3 + H)^+$ Calc'd: 560.2428, Found: 560.2427.

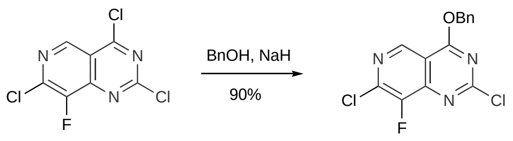

22

4-(benzyloxy)-2,7-dichloro-8-fluoropyrido[4,3-*d*]pyrimidine (**22**)
An oven-dried vial was charged with sodium hydride (60% dispersion in mineral oil, 1.0 g, 25.0 mmol), THF (12.5 mL), and a magnetic stir bar. A solution of benzyl alcohol (2.5 mL in 12.5 mL THF) was added dropwise at 23 ºC. In 10 min, gas evolution had subsided. The resulting cloudy mixture was allowed to settle for ~30 min, and the supernatant was used as a 1.0 M BnONa solution in THF. In a separate 100-mL flask, 2,4,7-trichloro-8-fluoro-pyrido[4,3-d]pyrimidine (2.50 g, 9.90 mmol, 1 equiv) was dissolved in THF (7.6 mL), and the resulting solution was cooled to 0 ºC. BnONa (1.0 M in THF, 17.0 mL, 1.70 equiv) was added via syringe, and the resulting mixture was stirred for 30 min at 0 ºC. The reaction mixture was partitioned between saturated aqueous sodium bicarbonate solution (50 mL) and ethyl acetate (50 mL). The layers were separated, and the aqueous layer was extracted with ethyl acetate (2 x 50 mL). The combined organic layers were dried over sodium sulfate. The dried solution was filtered, and the filtrate was concentrated. The residue was purified by column chromatography (0–30% ethyl acetate–hexanes, 80-g RediSep(R) Rf column, Teledyne ISCO, Lincoln, NE) to afford the product as a white solid (2.90 g, 90%).

^1H NMR (400 MHz, $CDCl_3$) δ 9.10 (s, 1H), 7.57 – 7.49 (m, 2H), 7.49 – 7.39 (m, 3H), 5.72 (s, 2H).
^{13}C NMR (100 MHz, $CDCl_3$) δ 167.93 (d, *J* = 2 Hz), 161.85, 148.20 (d, *J* = 270 Hz), 146.38 (d, *J* = 12 Hz), 143.64 (d, *J* = 8 Hz), 141.06 (d, *J* = 16 Hz), 133.89, 129.26, 129.03, 128.91, 111.70, 71.25.
^{19}F NMR (376 MHz, $CDCl_3$) δ -131.58.
HRMS $(C_{14}H_8Cl_2FN_3O + H)^+$ Calc'd: 324.0107, Found: 324.0267.

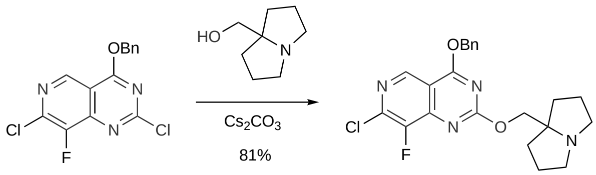

22 23

<u>4-(benzyloxy)-7-chloro-8-fluoro-2-((tetrahydro-1*H*-pyrrolizin-7a(5*H*)-yl)methoxy)pyrido[4,3-*d*]pyrimidine (**23**)</u>

An oven-dried 20-mL vial was charged with dichloride **22** (2.50 g, 7.71 mmol, 1 equiv), cesium carbonate (6.28 g, 19.3 mmol, 2.50 equiv), and a magnetic stir bar. 1,4-Dioxane (15.4 mL) and 1,2,3,5,6,7-hexahydropyrrolizin-8-ylmethanol (1.63 g, 11.6 mmol, 1.50 equiv) were added sequentially via syringe. The resulting mixture was heated to 60 ºC for 3 h. The reaction mixture was filtered through a 10-micron polyethylene funnel, and the filtrate and concentrated under reduced pressure. The residue was purified by column chromatography (0–20% methanol–dichloromethane + 0.2% ammonium hydroxide, 80-g RediSep(R) Rf column, Teledyne ISCO, Lincoln, NE) to afford the product as a white solid (2.69g, 81.3%).

^1H NMR (400 MHz, $CDCl_3$) δ 8.93 (s, 1H), 7.53 – 7.47 (m, 2H), 7.47 – 7.36 (m, 3H), 5.65 (s, 2H), 4.33 (s, 2H), 3.25 – 3.07 (m, 2H), 2.67 (dt, *J* = 10.2, 6.8 Hz, 2H), 2.06 (dt, *J* = 12.4, 6.1 Hz, 2H), 1.90 (p, *J* = 6.5 Hz, 4H), 1.69 (dt, *J* = 12.5, 7.5 Hz, 2H).
^{13}C NMR (100 MHz, $CDCl_3$) δ 169.03, 165.23, 149.63, 147.78, 147.67, 146.96, 143.12, 143.04, 140.07, 134.74, 128.85, 128.79, 128.56, 111.15, 74.30, 72.29, 70.05, 55.69, 36.04, 25.58.
^{19}F NMR (376 MHz, $CDCl_3$) δ -134.41.
HRMS $(C_{22}H_{22}ClFN_4O_2 + H)^+$ Calc'd: 429.1494, Found: 429.1590

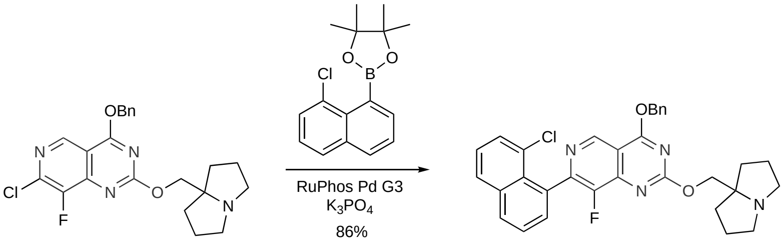

23 24

<u>4-benzyloxy-7-(8-chloro-1-naphthyl)-8-fluoro-2-(1,2,3,5,6,7-hexahydropyrrolizin-8-ylmethoxy)pyrido[4,3-d]pyrimidine (**24**)</u>

A 100-mL flask was charged with 4-benzyloxy-7-chloro-8-fluoro-2-(1,2,3,5,6,7-hexahydropyrrolizin-8-ylmethoxy)pyrido[4,3-d]pyrimidine (1.50 g, 3.50 mmol, 1 equiv.), 2-(8-chloro-1-naphthyl)-4,4,5,5-tetramethyl-1,3,2-dioxaborolane (1.51 g, 5.25 mmol, 1.50 equiv.) and

a magnetic stir bar. Toluene (5.8 mL) and an aqueous solution of potassium phosphate (1.50 M, 7.0 mL, 10.5 mmol, 3.00 equiv.) was added sequentially, giving rise to a biphasic mixture. A stream of argon was bubbled through the mixture for 5 min via a 20G needle. RuPhos Pd G3 (585 mg, 0.700 mmol, 0.200 equiv.) was added in one portion, the flask was fitted with a rubber septum, and the reaction mixture was heated to 60 ºC for 18 h. The reaction mixture was cooled to 23 ºC and then partitioned between water (20 mL) and ethyl acetate (20 mL). The layers were separated, and the aqueous layer was extracted with ethyl acetate (2 x 20 mL). The combined organic layers were washed with saturated sodium chloride solution, and the washed solution was dried over sodium sulfate. The dried solution was filtered, and the filtrate was concentrated. The residue was purified by column chromatography (0–30% methanol–dichloromethane, 40-g RediSep(R) Rf column, Teledyne ISCO, Lincoln, NE) to afford the product as a yellow powder (1.67 g, 86%).

^{1}H NMR (400 MHz, $CDCl_3$) δ 9.23 (s, 1H), 8.02 (dd, *J* = 8.0, 1.6 Hz, 1H), 7.89 (dd, *J* = 8.2, 1.3 Hz, 1H), 7.67 – 7.52 (m, 6H), 7.51 – 7.37 (m, 5H), 5.74 (s, 2H), 4.41 (s, 2H), 3.23 (dt, *J* = 11.0, 5.7 Hz, 2H), 2.71 (dt, *J* = 10.3, 6.8 Hz, 2H), 2.29 – 2.05 (m, 2H), 2.05 – 1.86 (m, 4H), 1.80 – 1.68 (m, 2H), .
^{13}C NMR (100 MHz, $CDCl_3$) δ 169.20, 164.87, 151.39, 149.83, 149.69, 147.83, 146.79, 142.60, 142.53, 135.93, 135.14, 131.76, 130.98, 130.62, 130.58, 129.38, 128.80, 128.76, 128.69, 128.56, 128.52, 128.35, 126.00, 125.56, 111.03, 73.95, 72.48, 69.75, 55.69, 55.67, 36.06, 36.04, 25.56, 25.54.
^{19}F NMR (376 MHz, $CDCl_3$) δ -138.14.
HRMS $(C_{32}H_{28}ClFN_4O_2 + H)^+$ Calc'd: 555.1963, Found: 555.1953.

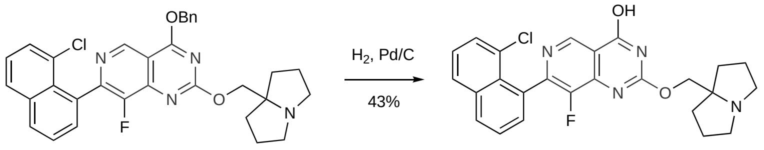

24 **25**

<u>7-(8-chloro-1-naphthyl)-8-fluoro-2-(1,2,3,5,6,7-hexahydropyrrolizin-8-ylmethoxy)pyrido[4,3-d]pyrimidin-4-ol (**25**)</u>

Argon was bubbled through a solution of 4-benzyloxy-7-(8-chloro-1-naphthyl)-8-fluoro-2-(1,2,3,5,6,7-hexahydropyrrolizin-8-ylmethoxy)pyrido[4,3-d]pyrimidine (2.50 g, 4.50 mmol, 1 equiv) in methanol (45 mL) for 5 min via a 20G needle. Palladium on carbon (10% wt, 479 mg, 0.450 mmol, 0.10 equiv.) was added carefully under a blanket of argon. The vial was fitted with a rubber septum, and a stream of hydrogen (balloon) was bubbled through the reaction mixture through a 22G x 4" needle. In 1 h, LC-MS analysis indicated full consumption of the starting material. The gas source was switched to argon and bubbling was continued for 5 min. The reaction mixture was filtered through a tightly packed pad of Celite under the protection of argon, and the filter cake was rinsed with methanol (2 x 10 mL). Care was taken not to allow the filter cake to become dry, and the filter cake was immediately moisturized with water upon completion of the filtration. The combined filtrate was concentrated under reduced pressure. The residue was purified by column chromatography (0–30% methanol–dichloromethane, 4-g RediSep(R) Rf column, Teledyne ISCO, Lincoln, NE) to afford the product as a yellow powder (890 mg, 42.5%).

^{1}H NMR (400 MHz, $CDCl_3$) δ 9.21 (s, 1H), 7.99 (dd, *J* = 6.7, 2.8 Hz, 1H), 7.87 (dd, *J* = 8.2, 1.3 Hz, 1H), 7.64 – 7.52 (m, 3H), 7.41 (t, *J* = 7.8 Hz, 1H), 4.47 (app q, *J* = 12.7 Hz, 2H), 3.64 – 3.49 (m, 2H), 3.04 – 2.89 (m, 2H), 2.29 – 2.03 (m, 6H), 1.98 – 1.84 (m, 2H).
^{19}F NMR (376 MHz, $CDCl_3$) δ -139.61.
^{13}C NMR (100 MHz, $CDCl_3$) δ 168.68, 162.34, 151.65, 149.89, 149.74, 149.08, 145.20, 145.09, 144.21, 144.15, 135.83, 132.22, 132.19, 131.00, 130.72, 130.17, 129.21, 128.69, 128.23, 125.77, 125.64, 116.97, 71.29, 55.51, 54.93, 35.19, 34.69, 24.90, 24.62.
HRMS ($C_{25}H_{22}ClFN_4O_2$ + H)$^+$ Calc'd: 465.1494, Found: 465.1518

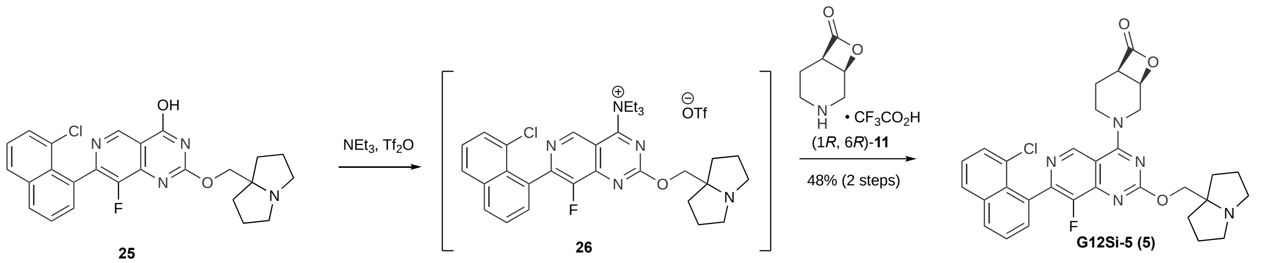

G12Si-5 (**5**).

A solution of **25** (50.0 mg, 0.107 mmol, 1 equiv.) in dichloromethane (0.27 mL) was cooled to 0 ºC, and triethylamine (45.0 µL, 0.323 mmol, 3.00 equiv.) and triflic anhydride (36.1 µL, 0.215 mmol, 2.00 equiv.) were added sequentially via syringe. In 1 h at 0 ºC, LC-MS analysis indicated that the starting material had been fully converted to the trimethylamine adduct **26**. The reaction mixture was directly concentrated under reduced pressure. DMF (0.27 mL) and triethylamine (45.0 µL, 0.323 mmol, 3.00 equiv.) were added to the residue, giving rise to a brown solution. (1*R*,6*R*)-**11** (25.9 mg, 0.107 mmol, 2.00 equiv.) was added as a solid. After stirring at 23 ºC for 1 h, the reaction mixture was diluted with 2.0 mL 1:1 acetonitrile:water + 5% formic acid. The resulting solution was loaded onto reverse-phase C18 column (Teledyne ISCO RediSep C18 30g Gold). Elution was performed with a linear gradient of 10–95% acetonitrile–water + 0.1% formic acid, and the product-containing fractions were lyophilized to afford the product as a yellow powder (31.0 mg, 48.3%).

^{1}H NMR (formate salt, 400 MHz, MeOD) δ 8.17 (dd, *J* = 8.2, 1.4 Hz, 1H), 8.04 (dd, *J* = 8.2, 1.3 Hz, 1H), 7.75 – 7.61 (m, 3H), 7.57 – 7.48 (m, 1H), 5.18 – 5.08 (m, 1H), 4.93 (dtd, *J* = 15.7, 6.3, 2.3 Hz, 1H), 4.69 (s, 2H), 4.36 (q, *J* = 7.1 Hz, 1H), 4.28 – 4.08 (m, 3H), 3.76 – 3.67 (m, 2H), 3.33 – 3.25 (m, 2H), 2.42 – 2.30 (m, 4H), 2.30 – 2.03 (m, 5H), 1.43 – 1.29 (m, 2H).
^{19}F NMR (formate salt, 376 MHz, MeOD) δ -139.57, -139.90.
^{13}C NMR (formate salt, 100 MHz, MeOD) δ 170.17, 164.48, 163.18, 144.08, 144.01, 136.00, 135.95, 130.90, 130.76, 129.88, 129.26, 128.47, 128.24, 126.08, 125.48, 125.43, 121.77, 118.83, 111.03, 80.32, 69.59, 68.97, 55.26, 46.42, 45.91, 45.69, 34.00, 23.86, 19.63, 19.54, 7.59.
Note: Product appears as a mixture of conformational isomers. NMR peaks were reported as seen.
HRMS ($C_{31}H_{29}ClFN_5O_3$ + H)$^+$ Calc'd: 574.2021, Found: 574.2015.
