# Supplementary material for: Chemical acylation of an acquired serine suppresses oncogenic signaling of K-Ras(G12S)
Source: Nat Chem Biol. 2022 Jul 21;18(11):1177–83. doi: 10.1038/s41589-022-01065-9 (PMC9596369; doi:10.1038/s41589-022-01065-9)
Supplement: Supplementary file 2 — Reporting Summary [file 41589_2022_1065_MOESM2_ESM.pdf]

nature portfolio

Corresponding author(s): Kevan Shokat

Last updated by author(s): May 9, 2022

Reporting Summary

Nature Portfolio wishes to improve the reproducibility of the work that we publish. This form provides structure for consistency and transparency in reporting. For further information on Nature Portfolio policies, see our Editorial Policies and the Editorial Policy Checklist.

Statistics

For all statistical analyses, confirm that the following items are present in the figure legend, table legend, main text, or Methods section.

n/a	Confirmed	
☐	☒	The exact sample size (*n*) for each experimental group/condition, given as a discrete number and unit of measurement
☐	☒	A statement on whether measurements were taken from distinct samples or whether the same sample was measured repeatedly
☐	☒	The statistical test(s) used AND whether they are one- or two-sided<br>*Only common tests should be described solely by name; describe more complex techniques in the Methods section.*
☒	☐	A description of all covariates tested
☒	☐	A description of any assumptions or corrections, such as tests of normality and adjustment for multiple comparisons
☐	☒	A full description of the statistical parameters including central tendency (e.g. means) or other basic estimates (e.g. regression coefficient) AND variation (e.g. standard deviation) or associated estimates of uncertainty (e.g. confidence intervals)
☒	☐	For null hypothesis testing, the test statistic (e.g. *F*, *t*, *r*) with confidence intervals, effect sizes, degrees of freedom and *P* value noted<br>*Give P values as exact values whenever suitable.*
☒	☐	For Bayesian analysis, information on the choice of priors and Markov chain Monte Carlo settings
☒	☐	For hierarchical and complex designs, identification of the appropriate level for tests and full reporting of outcomes
☒	☐	Estimates of effect sizes (e.g. Cohen's *d*, Pearson's *r*), indicating how they were calculated

Our web collection on statistics for biologists contains articles on many of the points above.

Software and code

Policy information about availability of computer code

Data collection	NMR: Bruker Topspin 3.1<br>Western Blot: LICOR Odyssey 2.1<br>Plate reader: TECAN SparkControl 2.1<br>LC-MS: Waters MassLynx 4.2<br>Fluorescence scanner: GE Typhoon FLA 9000
Data analysis	GraphPad Prism 9.0<br>MestroNova 14.1.2<br>ImageJ 2.3.0<br>MaxQuant 2.0.3.1<br>CCP4i2 1.0.2 (including iMosflm 7.4.0, Aimless 0.7.4 and Phaser 2.8.3 modules)<br>Coot 0.9.6<br>Phenix 1.19.2

For manuscripts utilizing custom algorithms or software that are central to the research but not yet described in published literature, software must be made available to editors and reviewers. We strongly encourage code deposition in a community repository (e.g. GitHub). See the Nature Portfolio guidelines for submitting code & software for further information.

Data

Policy information about availability of data

All manuscripts must include a data availability statement. This statement should provide the following information, where applicable:

- Accession codes, unique identifiers, or web links for publicly available datasets
- A description of any restrictions on data availability
- For clinical datasets or third party data, please ensure that the statement adheres to our policy

Atomic coordinates and structure factors for the reported crystal structures have been deposited with the Protein Data Bank (PDB), with the following accession numbers: K-Ras(G12S)•GDP – 7TLK; K-Ras(G12S)•GDP•1 – 7TLE; K-Ras(G12S)•GDP•5 – 7TLG. Uncropped, unprocessed gel images are provided as Source Data files accompanying this paper.

Field-specific reporting

Please select the one below that is the best fit for your research. If you are not sure, read the appropriate sections before making your selection.

☒ Life sciences ☐ Behavioural & social sciences ☐ Ecological, evolutionary & environmental sciences

For a reference copy of the document with all sections, see nature.com/documents/nr-reporting-summary-flat.pdf

Life sciences study design

All studies must disclose on these points even when the disclosure is negative.

Sample size	For plate reader based assays, three technical replicates were performed in each experiment. All experiments have been performed in at least two biological replicates (see legends for numbers of biological replicates for each experiment)
Data exclusions	No data was excluded from analyses.
Replication	All attempts at replication were successful (see figure legends for the number of replicates for each experiment). We did not replicate X-ray crystallography experiments.<br>Response to editor's inquiry as to why replication was not performed for X-ray crystallography experiments:<br>Crystal nucleation and growth has a sporadic nature and the size, quality and shape of the crystal do not easily replicate even under identical conditions. The protein crystals used in this study are obtained from hanging drop screens. X-ray diffraction of the said crystals cannot be replicated on the same crystal because samples degrade under the irradiation of the X-ray beam. It is common practice to use a single X-ray diffraction dataset for structure solution. We report the growth condition, diffraction condition and structural refinement parameters for each structure reported.
Randomization	N/A. Each biochemical experiment in this study is rationally designed and leads to a specific conclusion. Samples were not randomized.
Blinding	N/A. Each biochemical experiment in this study is rationally designed and leads to a specific conclusion. Samples were not blind.

Reporting for specific materials, systems and methods

We require information from authors about some types of materials, experimental systems and methods used in many studies. Here, indicate whether each material, system or method listed is relevant to your study. If you are not sure if a list item applies to your research, read the appropriate section before selecting a response.

Materials & experimental systems

n/a	Involved in the study	
☐	☒	Antibodies
☐	☒	Eukaryotic cell lines
☒	☐	Palaeontology and archaeology
☒	☐	Animals and other organisms
☒	☐	Human research participants
☒	☐	Clinical data
☒	☐	Dual use research of concern

Methods

n/a	Involved in the study	
☒	☐	ChIP-seq
☒	☐	Flow cytometry
☒	☐	MRI-based neuroimaging

Antibodies

Antibodies used	P-AKT [S473] Cell Signaling Technology 4060, Clone D9E<br>P-AKT [T308] Cell Signaling Technology 4056, Clone 244F9<br>AKT Cell Signaling Technology 2920, Clone 40D4

P-ERK [T202/Y204] Cell Signaling Technology 9101, Polyclonal
Total ERK Cell Signaling Technology 4695, Clone 137F5
Pan-Ras Abcam 108602, Clone EPR3255
Ras(G12S) NewEast Bio 26186, Clone# Not Available
GAPDH Proteintech 60004-1-Ig, Clone 1E6D9
goat anti-rabbit IgG-IRDye 800, LI-COR, 926-32211, Clone# Not Available
goat anti-mouse IgG-IRDye 680, LI-COR, 926-68070, Clone# Not Available

Validation

The validation of the Ras(G12S) antibody is included in the Supplementary Information. All other commercial antibodies have been validated by the manufacturers (see website links below)
P-AKT [S473]: https://www.cellsignal.com/products/primary-antibodies/phospho-akt-ser473-d9e-xp-rabbit-mab/4060
P-AKT [T308]: https://www.cellsignal.com/products/primary-antibodies/phospho-akt-thr308-244f9-rabbit-mab/4056
AKT: https://www.cellsignal.com/products/primary-antibodies/akt-pan-40d4-mouse-mab/2920
P-ERK [T202/Y204]: https://www.cellsignal.com/products/primary-antibodies/phospho-p44-42-mapk-erk1-2-thr202-tyr204-antibody/9101
Total ERK: https://www.cellsignal.com/products/primary-antibodies/p44-42-mapk-erk1-2-137f5-rabbit-mab/4695
Pan-Ras: https://www.abcam.com/ras-antibody-epr3255-ab108602.html
GAPDH: https://www.ptglab.com/products/GAPDH-Antibody-60004-1-Ig.htm
Goat anti-rabbit IgG-IRDye 800: https://www.licor.com/documents/rfm2hw40wf33p06f3ndjrcorwi5usbft
Goat anti-mouse IgG-IRDye 680: https://www.licor.com/documents/7bohf1sfzugccz22fh0um00cvz8ocizf

Eukaryotic cell lines

Policy information about cell lines

Cell line source(s)

A549: UCSF Cell Culture Facility
HKA1: Japanese Collection of Research Biosources Cell Bank (JCRB)
KMS20: Japanese Collection of Research Biosources Cell Bank (JCRB)
LS123: American Type Culture Collection (ATCC)
H358: American Type Culture Collection (ATCC)
A375: American Type Culture Collection (ATCC)
SW1990: American Type Culture Collection (ATCC)
Ba/F3: German Collection of Microorganisms and Cell Cultures GmbH (DSMZ)
EcoPack 293: Clonetech

Authentication

Cell lines from JRCB, DSMZ and ATCC were STR profiled by the manufacturer.
A549 cells were STR profiled by the UCSF Cell Culture Facility.
EcoPack 293 is a commercial cell line established and maintained by the manufacturer (Clonetech) and authentication is provided by the manufacturer.

Mycoplasma contamination

All cell lines were tested mycoplasma negative using MycoAlert™ Mycoplasma Detection Kit (Lonza).

Commonly misidentified lines
(See ICLAC register)

None found.
