# Supplementary figures and images for: Chemical acylation of an acquired serine suppresses oncogenic signaling of K-Ras(G12S)
Source: Nat Chem Biol. 2022 Jul 21;18(11):1177–83. doi: 10.1038/s41589-022-01065-9 (PMC9596369; doi:10.1038/s41589-022-01065-9)

# Uncropped gel images for Fig. 1a

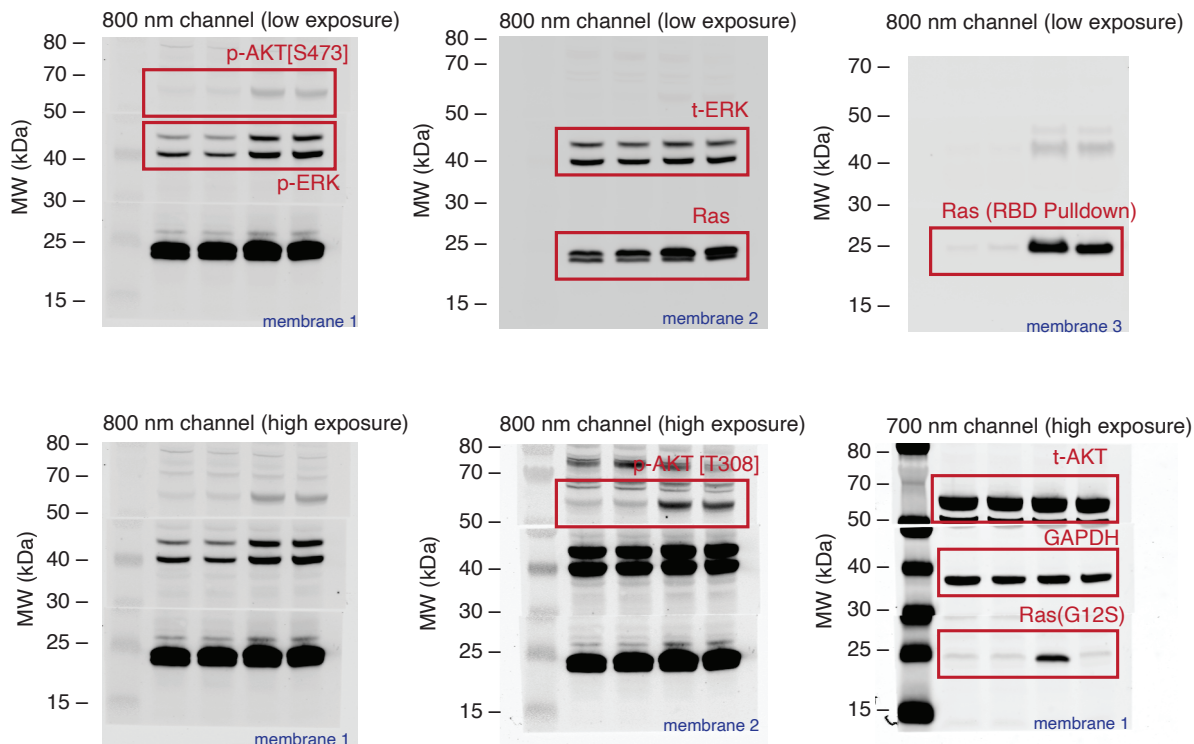

Supplement: Source Data Fig. 1 — Unprocessed western blots for Fig. 1. [file 41589_2022_1065_MOESM4_ESM.pdf]

## Uncropped gel images for Extended Data Fig. 6c

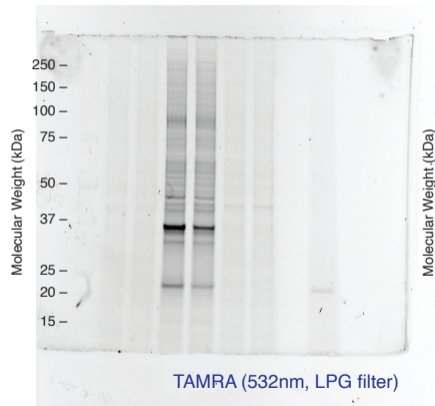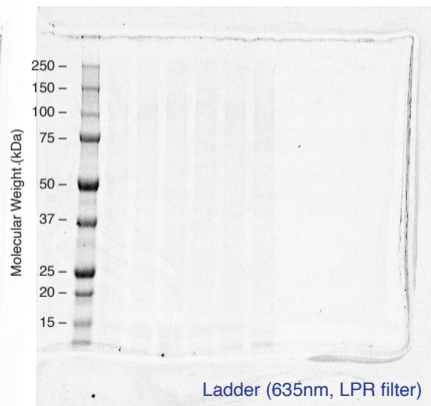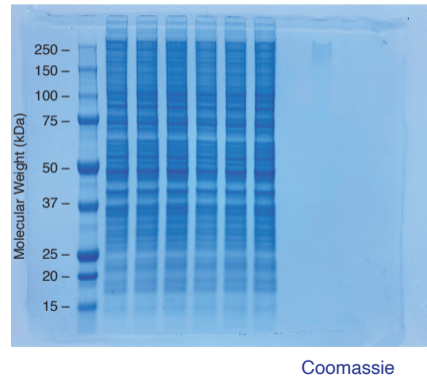

Supplement: Source Data Extended Data Fig. 6 — Unprocessed gel images for Extended Data Fig. 6. [file 41589_2022_1065_MOESM6_ESM.pdf]
