# Supplementary material for: Chemical acylation of an acquired serine suppresses oncogenic signaling of K-Ras(G12S)
Source: Nat Chem Biol. 2022 Jul 21;18(11):1177–83. doi: 10.1038/s41589-022-01065-9 (PMC9596369; doi:10.1038/s41589-022-01065-9)
Supplement: Source Data Fig. 4 — Unprocessed western blots for Fig. 4. [file 41589_2022_1065_MOESM5_ESM.pdf]

Uncropped gel images for Fig. 4b

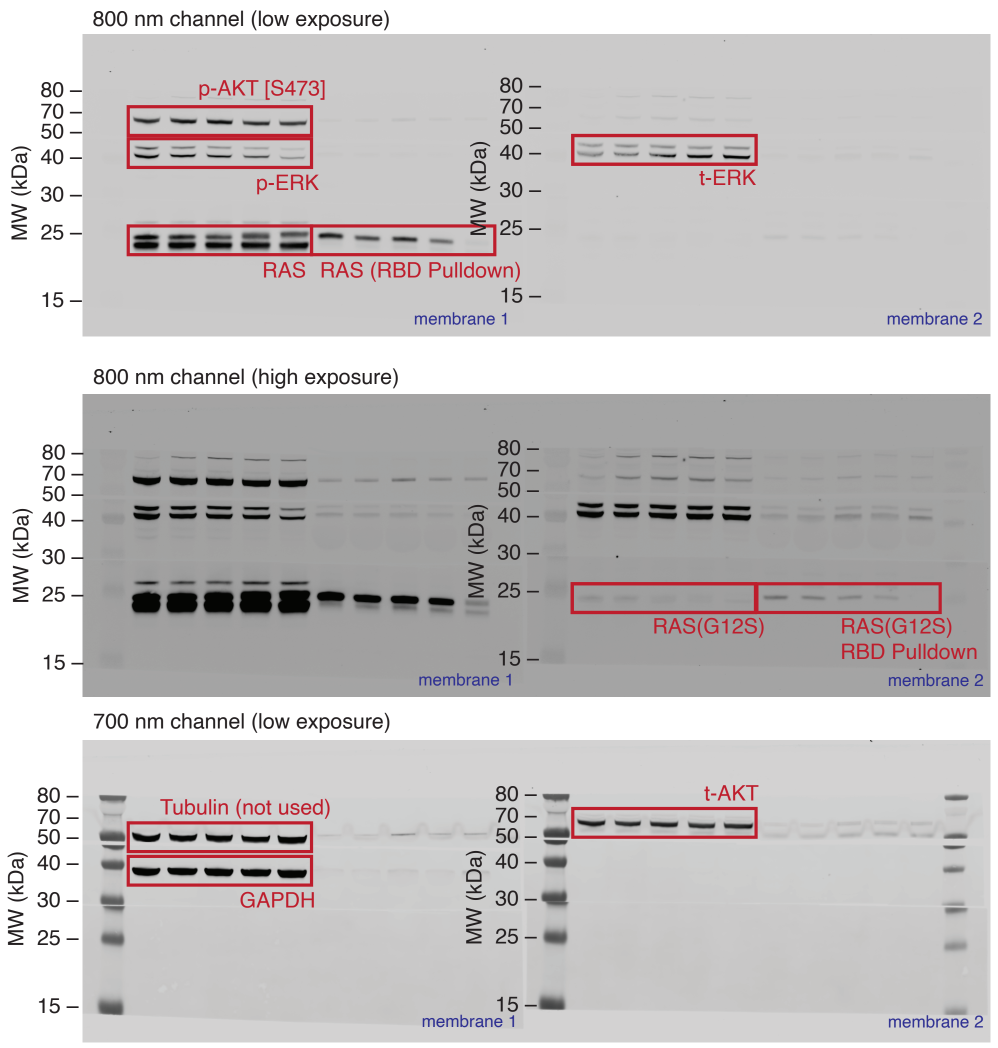

Uncropped gel images for Fig. 4d

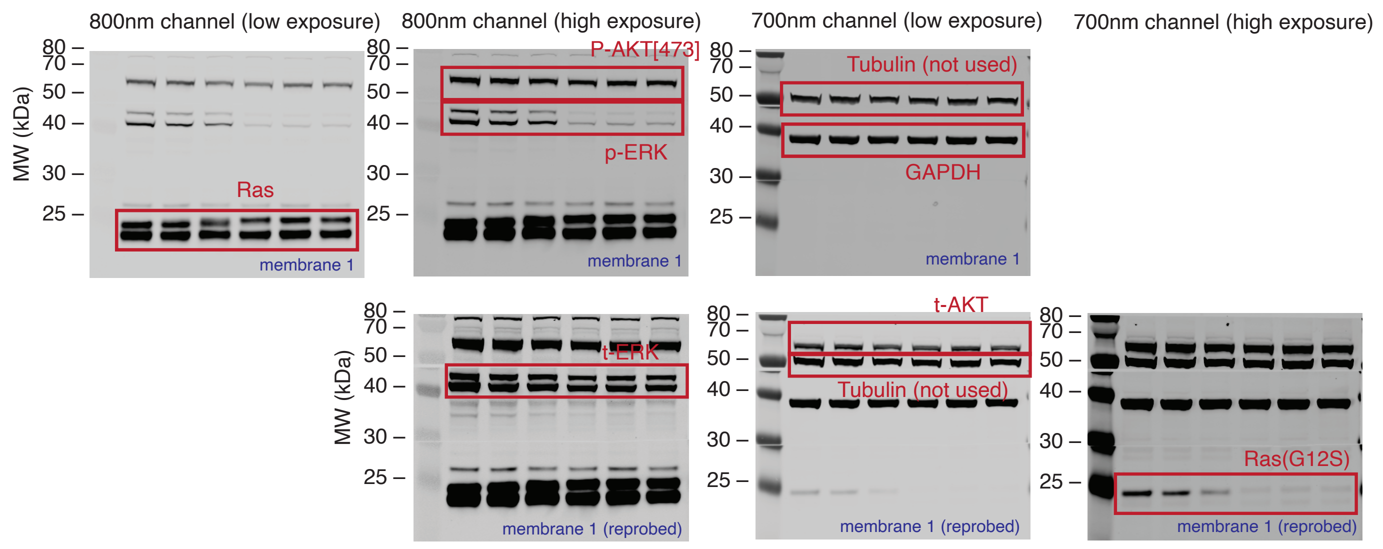

Uncropped gel images for Fig. 4e

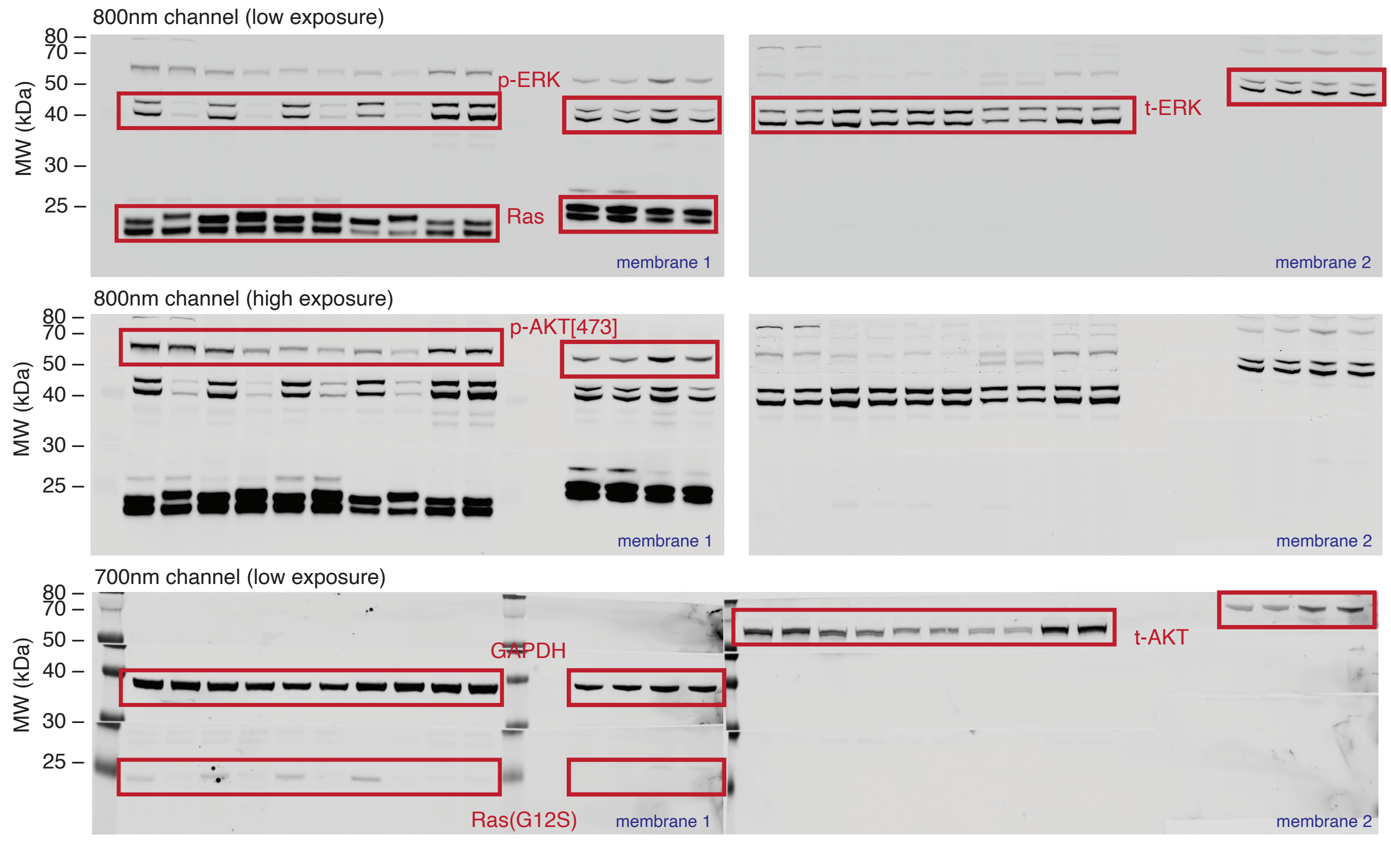

Note: Due to limitations of gel size (12-well), the samples (1-10 and 11-14) were run on two separate gels and transferred onto a single membrane in a single transfer sandwich. Some bands have vertical offsets between the two gels due to technical difficulties of perfectly aligning gels during the transfer.
